# Supplementary material for: Vertebrate host phylogeny influences gut archaeal diversity
Source: Nat Microbiol. 2021 Oct 26;6(11):1443–54. doi: 10.1038/s41564-021-00980-2 (PMC8556154; doi:10.1038/s41564-021-00980-2)
Supplement: Supplementary file 1 — Supplementary Figs. 1–34 and figure captions, captions for Tables 1–12, Results and raw gel image for S26. [file 41564_2021_980_MOESM1_ESM.pdf]

nature portfolio

https://doi.org/10.1038/s41564-021-00980-2

Supplementary information

Vertebrate host phylogeny influences gut archaeal diversity

In the format provided by the authors and unedited

Supplemental: Vertebrate host phylogeny influences gut archaeal diversity

Supplemental Results

Prevalence and diversity of Archaea *across vertebrate clades*

Of 311 genomic DNA samples from 5 vertebrate taxonomic classes, 185 (60%) passed 16S rRNA gene PCR amplification, MiSeq sequencing, and sequence data quality control (Table S2). Success rates were highest for *Reptilia* (73%) and *Aves* (67%), 58% for *Mammalia*, 50% for *Amphibia*, and 50% for *Reptilia* (Figure S2). The 185 successful samples comprised mostly wild individuals (76%) and a total of 110 species, with a mean 1.7 ± 4.3 s.d. samples per species (Figure S1). *Mammalia* made up the majority of samples (72%); still, non-mammalian samples spanned 22 families and 35 genera. In regards to diet, success rates were 70, 56, and 46% for herbivores, omnivores, and carnivores, respectively (Figure S2). Feces samples had a substantially higher success rate (62%) versus gut contents (38%), but there was little difference between wild and captive individuals (62 versus 56%, respectively). The mean per-species success rate was 61% ± 49 s.d., and when just assessing species with >1 sample (72 of 158), the success rate was 63% ± 37.3 s.d. Plotting the number of successful and failed samples onto a phylogeny of all species showed that success often varied among individuals of a species (Figure S4). In addition, some phylogenetic clustering of success rates could be observed. Indeed, when just considering *Mammalia*, which made up the majority of samples (73%), the orders *Lagomorpha*, *Carnivora*, and *Rodentia* had the lowest success rates (<50% for each), while success rates were 100% for *Monotremata*, *Perissodactyla*, and *Proboscidea* (Figure S2). While these findings are compelling, one must consider that failure may have resulted from many phenomena besides absence of *Archaea* from the gut, such as PCR inhibitors or insufficient DNA for effective amplification. Still, success across highly varied host taxonomic groups, diets, and sample types indicates that Archaea are widespread among vertebrates, regardless of diet.

Rarefaction analysis using the Shannon index revealed that archaeal diversity saturated at a low sampling depth of approximately 250 sequences, regardless of the host class (Figure S7A). We confirmed these results with another rarefaction method that extrapolates diversity beyond obtained sampling depths, with diversity based on Hill numbers (Figure S7B). These results contrast most gut microbiome studies using the commonly used "universal" Earth Microbiome 16S rRNA primer set 515F-806R, in which bacterial and archaeal diversity is usually not saturated for the sampling depths reached Walters et al., "Improved Bacterial 16S rRNA Gene (V4 and V4-5) and Fungal Internal Transcribed Spacer Marker Gene Primers for Microbial Community Surveys"; Thompson et al., "A Communal Catalogue Reveals Earth's Multiscale Microbial Diversity"; Youngblut et al., "Host Diet and Evolutionary History Explain Different Aspects of Gut Microbiome Diversity among Vertebrate Clades.".

The dataset comprised 1891 amplicon sequence variants (ASVs), with a rather diverse taxonomic composition for *Archaea*, comprising 6 phyla (*Asgardaeota*, *Crenarchaeota*, *Diapherotrites*, *Euryarchaeota*, *Nanoarchaeaeota*, *Thaumarchaeota*) and 10 classes (Figure 1). Class-level taxonomic compositions were fairly consistent among individuals of each host

species (Figure S8; Table S4). *Methanobacteria* ASVs were generally most prevalent across host species (mean of ~4%; Figure S6). We note that *Asgardarchaeota* and *Diapherotrites* were each only represented by 1 ASV, and each were found in only 1 species: *Asgardaeota* in the the European Otter (*Lutra lutra*) and *Diapherotrites* in the Smooth Newt (*Lissotriton vulgaris*). Neither clade is known to be animal-associated Borrel et al., "The Host-Associated Archaeome.". Also, the *Thermococci* class (*Euryarchaeota* phylum) comprised only 2 ASVs, with one only found in the Common Carp (*Cyprinus carpio*) and the other in the European Otter. Both ASVs were classified as *Methanofastidiosales*, with one identified as *Methanofastidiosum*. No member of this class is known to be host-associated Söllinger and Urich, "Methylotrophic Methanogens Everywhere - Physiology and Ecology of Novel Players in Global Methane Cycling"; Borrel et al., "The Host-Associated Archaeome.". Plotting mean relative abundances of taxonomic classes onto a tree of all species revealed that *Methanobacteria* (*Euryarchaeota* phylum) dominated in many species, but dramatically different microbiome compositions were observed scattered across the phylogeny. For instance, *Thermoplasmata* (*Euryarchaeota* phylum) dominated in multiple non-human primates, while two *Mammalia* and one *Aves* species were nearly completely comprised of *Nitrososphaeria* (*Thaumarchaeaota* phylum): the European badger (*Meles meles*), the Western European Hedgehog (*Erinaceus europaeus*), and the Rook (*Corvus frugilegus*). *Halobacteria* (*Euryarchaoeota* phylum) dominated the Goose (*Anser anser*) microbiome, which were all sampled from salt marshes. The class was also noticeably present in some distantly related animals inhabiting high salinity biomes (*e.g.,* the Nile Crocodile and the Short Beaked Echidna; Tables S1 & S4). *Bathyarchaeia*, a class in the *Crenarchaea* phylum according to the SILVA database taxonomy but also known as the Candidatus *Bathyarchaeota* phylum, are not known to inhabit the vertebrate gut Ibid.; however, we observed a total of 9 *Bathyarchaeia* ASVs in 8 samples, comprising 6 species spanning 4 taxonomic classes (all except *Mammalia*; Table S5). Most of these ASVs were detected in only one sample apiece, but 2 *Bathyarchaeia* ASVs were both observed in each of the two Smooth Newt samples (Figure S6). The total *Bathyarchaeia* ASVs relative abundance was <0.5% in 4 of the species, while substantially higher (3.3%) in the Nile Crocodile (*Crocodylus niloticus*), and quite abundant in 2 Smooth Newt samples (17.9 and 42.2%).

Only 40% of ASVs had a ≥97% sequence identity match (a pseudo-species level) to any cultured representative in the All Species Living Tree database (Figure S10A). Of the 10 taxonomic classes represented by all ASVs, 5 had no match at ≥85% sequence identity: *Odinarchaeia*, *Bathyarchaeia*, *Iainarchaeia*, *Woesarchaeia*, and *Thermococci*. Taxonomic novelty to cultured representatives differed substantially among the other 5 classes (Figure S10B); only *Methanobacteria* had >50% ASVs with a species-level match (52%), while <20% of ASVs belonging to *Thermoplasmata* and *Nitrososphaeria* had such a match. These findings suggest that our dataset consists of a great deal of uncultured taxonomic diversity.

Archaea-*targeting primers reveal much greater archaeal diversity*

We compared the archaeal diversity identified with the archaeal-targeting primers ("16S-arc") used in this study to the standard "universal" 16S rRNA primers ("16S-uni") used by Youngblut and colleagues on many of the same samples Youngblut et al., "Host Diet and Evolutionary History Explain Different Aspects of Gut Microbiome Diversity among Vertebrate Clades.". Importantly, both datasets were processed in the same manner (see Methods). A total

of 140 samples overlapped between the two datasets, with the majority of species (77%) consisting of mammals, but all 5 classes were represented (Figure S11). The 16S-uni primers generated a total of 169 ASVs, which is only 12.1% of archaeal ASVs generated by the 16S-arc primers for the same samples. All archaeal classes except the *Soil Crenarchaeal Group* were substantially more represented in the 16S-arc dataset, with 6 classes completely absent from the 16S-uni dataset: *Nitrososphaeria*, *Woesarchaeia*, unclassified *Eukyarchaeota*, *Iainarchaeia*, *Bathyarchaeia*, and *Odinarchaeia*. Besides the *Soil Crenarchaeal Group*, class-level prevalence across host species was substantially higher across hosts when grouped by taxonomic class or diet (Figure S11). For example, *Methanobacteria* was observed in all host species via the 16S-arc primers, while prevalence dropped substantially for 16S-uni primers (*e.g.,* only 9% for *Aves*). These findings show that the “universal” NGS 16S rRNA primers used for most microbiome studies can substantially undersample archaeal diversity, as previously observed Raymann et al., “Unexplored Archaeal Diversity in the Great Ape Gut Microbiome”; Koskinen et al., “First Insights into the Diverse Human Archaeome: Specific Detection of Archaea in the Gastrointestinal Tract, Lung, and Nose and on Skin”; Pausan et al., “Exploring the Archaeome: Detection of Archaeal Signatures in the Human Body.”

Host diet and evolutionary history explain various aspects of archaeal diversity

We used multiple regression on matrices (MRM) to assess which potential factors explain archaeal beta diversity. We employed this approach because archaeal beta diversity, host phylogenetic relatedness, and geographic distance can be inherently represented as distance matrices, while distances can be calculated for other explanatory factors such as similarity of detailed diet compositions (see Methods). Due to a lack of within-species phylogenetic relatedness data, we used one individual per host species and assessed intra-species variation by repeating the analysis 99 more times, each time with one randomly selected individual per species. Unless otherwise noted, this permutation-based intra-species sensitivity analysis was used for all hypothesis testing.

Geographic distance, habitat, and technical components (*e.g.,* feces versus gut contents) did not significantly explain beta diversity, regardless of the diversity metric (Figure 2A). Host phylogeny significantly explained diversity as measured by unweighted UniFrac, Bray-Curtis, and Jaccard; however, significance was not quite reached for weighted UniFrac. The percent variation explained was dependent on the beta diversity measure and varied from ~28% for Jaccard to ~12% for unweighted UniFrac. In contrast to host phylogeny, diet was only explanatory for Bray-Curtis, with ~12% of variance explained. Mapping the major factors onto ordinations qualitatively supported our results (Figure S12). Applying the same MRM analysis to just non-mammalian species did not generate any significant associations between host phylogeny or diet (Figure S14), likely due to the low sample sizes (n = 39). However, host phylogeny did have comparable coefficients as when including all species and were nearly significant for both the Bray-Curtis and Jaccard indices, while diet showed no such trend towards significance. These findings suggest that host evolutionary history mediates vertebrate gut archaeal diversity more than diet, with diet mainly altering the abundances of archaeal ASVs shared by various hosts, while host phylogeny also alters the composition of archaeal taxa.

We also assessed alpha diversity via MRM in order to provide a consistent comparison to our beta diversity assessment, with alpha diversity represented here as a euclidean distance

matrix (Figure S15). In contrast to beta diversity, no factors significantly explained alpha diversity calculated via either the Shannon Index or Faith's Phylogenetic Diversity (Faith's PD). Of note, geographic distance nearly significantly explained Shannon Index diversity (P = 0.06), while the same was true of habitat for Faith's PD (P = 0.16).

A signal of Archaea-Vertebrata *co-phylogeny*

To test for corresponding phylogenetic associations on both the host phylogeny and the archaeal 16S rRNA phylogeny, we employed two approaches to quantify signals of co-phylogeny: Procrustes Application to Cophylogenetic Analysis (PACo) and ParaFit Paradis, Claude, and Strimmer, "APE: Analyses of Phylogenetics and Evolution in R Language"; Hutchinson et al., "Paco: Implementing Procrustean Approach to Cophylogeny in R.". Both PACo and ParaFit tests were both significant (P < 0.01) for each of the 100 permutations of subsampling one individual per host species, indicating a signal of co-phylogeny that is robust to intra-species microbiome variation. We investigated which host species showed the strongest signal of cophylogeny by assessing the distribution of PACo Procrustes residuals, which provide an indication of local congruence between phylogenies (lower residuals indicate a stronger congruence). *Mammalia* showed a substantially stronger association relative to the other four classes (Figure 2D), with residuals decreasing in the order of *Actinopterygii* > *Amphibia* > *Reptilia* > *Aves* ≫ *Mammalia*, and these differences were significant (Kruskal-Wallis < 0.01; pairwise Wilcox < 0.01 for all). In regards to diet, residuals were significantly lower for herbivores relative to omnivores and carnivores (Wilcox, P < 0.0001), while carnivores and omnivores did not significantly differ (Figure 2E).

Specific archaeal ASVs are associated with host phylogeny

Given the evidence of host phylogeny explaining aspects of archaeal gut microbiome diversity, we sought to further resolve this association by testing whether archaeal taxon abundance is clustered on the host phylogeny. We found 37 ASVs to show significant global phylogenetic signal (Pagel's λ, adj. P < 0.05) spanning three phyla: *Euryarchaota*, *Thaumarchaeota*, and *Crenarchaeota* (Figure 2C). The clade with the highest number of significant ASVs (n = 15) was *Methanobacteriaceae*, followed by *Nitrososphaeraceae* (n = 12), and *Methanocorpusculaceae* (n = 5). While lambda coefficients varied across ASVs, most showed a very strong association (Pagel's λ > 0.9), with major exceptions being a *Methanosarcinaceae* ASVs and an unclassified *Methanomicrobia* ASV (Figure 2C).

We next tested for local phylogenetic signals to resolve archaeal taxon specificities for particular host clades. We used the local indicator of phylogenetic association (LIPA) and found 25 ASVs to have significant associations with certain host clades. Mapping significant associations on the host phylogeny revealed that clade-specificity was generally shallow and often spanned only 2 species (Figure S17). For instance, 4 *Nitrososphaeraceae* ASVs were associated with 2 snake species (*Zamenis longissimus* and *Natrix natrix*), 3 *Methanobrevibacter* ASVs were associated with 2 species of kangaroo (*Macropus giganeus* and *Macropus fuliginosus*), and a *Methanocorpusculum* ASV was associated with both camel species (*Camelus dromedarius* and *Camelus bactrianus*). The 2 major exceptions to this trend were the *Methanothermobacter* ASVs, which associated with many species of Aves, while the

Methanobrevibacter and *Methanosphaera* ASVs associated with many *Artiodactyla* species (true ruminants; Figure S17). Summarizing the number microbe-host clade associations revealed clear partitioning of archaeal taxa by host clade, except for *Methanobrevibacter*, for which at least one ASV was associated with each host order for which any phylogenetic signal was observed (n = 23; Figure S17B). Altogether, these results help to resolve which particular archaeal clades are most strongly associated with host evolutionary history.

We also tested for phylogenetic signal of alpha diversity but found no significant global associations when measuring diversity via the Shannon Index or Faith's PD ($P > 0.05$) and no local associations (adj. $P > 0.05$). These findings correspond with our MRM analysis of alpha diversity in that host phylogenetic relatedness does not seem to correspond with total archaeal diversity in the gut.

Specific methanogen ASVs are associated with diet

We used two methods to resolve the specific effects of diet on the archaeal microbiome while controlling for host evolutionary history: phylogenetic generalized least squares (PGLS) and randomization of residuals in a permutation procedure (RRPP). The former is a common test for association between traits while controlling for phylogenetic relatedness, while the latter can exhibit higher statistical power while minimizing false positives Revell, "Phylogenetic Signal and Linear Regression on Species Data: Phylogenetic Regression"; Collyer and Adams, "RRPP: An R Package for Fitting Linear Models to High‐dimensional Data Using Residual Randomization.". PLGS identified 10 ASVs as being significantly associated with diet (adj. $P < 0.05$; Figure S16). All ASVs belonged to the *Euryarchaeota* phylum, and comprised 4 genera: *Methanobrevibacter*, *Methanosphaera*, *Methanothermobacter*, and candidatus *Methanomethylophilus*. The RRPP analysis identified the same 10 ASVs along with 5 more that belonged to the same genera (Figure 2B). We used the RRPP models to predict ASV abundances with 95% confidence intervals (CIs) for each diet in order to determine diet-specific enrichment. *Methanobacteria* ASVs differed in their responses to diet, with 5 being most abundant in herbivores, while the other 6 were more abundant in omnivores/carnivores (Figure 2B). Notably, diet enrichment differed even among ASVs belonging to the same genus. In contrast to the *Methanobacteria* ASVs, all 4 *Methanomethylophilus* ASVs were predicted as more abundant in omnivores/carnivores. These findings suggest that diet influences the abundances of particular ASVs, and even closely related ASVs can have contrasting associations to diet. All significant ASVs were methanogens, which may be due to the species studied (*e.g.,* a mammalian bias) or possibly because certain methanogens respond readily to diet, possibly due to syntrophic associations with diet-specific bacteria.

When applied to alpha or beta diversity, neither PGLS nor RRPP identified any significant associations with diet after accounting for host phylogenetic relatedness. These findings correspond with our MRM analyses by indicating that diet is not a strong moduator of overall archaeal diversity in the vertebrate gut, although certain ASVs do seem to be substantially affected (Figures 2B & S16).

Evidence of widespread Methanobacteria *presence in the ancestral vertebrate gut*

We utilized ancestral state reconstruction (ASR) to investigate which archaeal clades were likely present in the ancestral vertebrate gut. Traits were defined as archaeal taxon abundances. Notably, we used a method that incorporated intra-species trait variance, allowing us to directly utilize the entire host dataset for the reconstruction (see Methods). Our model for predicting class-level abundances was overall quite accurate at extant species trait prediction (adj. $R^2 = 0.86$, $P < 2e\text{-}16$; Figure S19). However, predictions were not accurate for 2 of the 6 classes (*Halobacteria* and *Nitrososphaeria*, $P > 0.1$), likely due to low prevalence across extant host species (Figures 2 & S20). Excluding the poorly predicted classes, the 95% CIs for predicted abundances were constrained enough to be informative (mean of 26% ± 29 s.d.) across extant and ancestral host species. The model revealed that *Methanobacteria* was uniquely pervasive across ancestral nodes, while other classes were sparsely distributed among extant taxa and across a few, more recent ancestral nodes (Figures 2 & S20). Moreover, the model predicted that *Methanobacteria* was the only class to be present in the last common ancestor (LCA) of all mammals and the LCA of all 5 host taxonomic classes (Figure 3B & 3C).

We also generated an ASR model for genus-level abundances of all genera in the *Methanobacteria* class in order to resolve the association between *Methanobacteria* clades and the ancestral vertebrate gut. Our model was somewhat more accurate at predicting extant traits than our class-level model ($R^2 = 0.93$, $P < 2e\text{-}16$; Figure S19), and all 4 genera were accurately predicted ($P < 5.5e\text{-}10$ for all). Predicted trait value 95% CIs were again informative (mean of 28 ± 24 s.d.). The model predicted 3 of the 4 genera to be present in the LCA of all mammals and the LCA of all host species (Figure 3F & 3G). Of the 3, *Methanobrevibacter* and *Methanothermobacter* were predicted to have similar abundances for both LCAs (~30-35%), while *Methanosphaera* was much lower (~5%). Mapping predicted abundances onto the host phylogeny revealed that *Methanobrevibacter* was predicted as most highly abundant in the *Artiodactyla* and generally abundant across most mammalian clades (Figure S21). In contrast, *Methanothermobacter* was predicted to be most highly abundant and prevalent across the avian and also mammalian clades in which *Methanobrevibacter* was less abundant (*e.g., Carnivora* and *Rodentia*). *Methanosphaera* was predicted to be prevalent across most animal clades, but generally at low abundance.

Importantly, we found both ASR analyses (class and genus levels) to be robust to biases in the number of samples per host species (Figure S22).

Methanothermobacter *abundance is correlated with body temperature*

Methanothermobacter is not known to be host-associated Borrel et al., “The Host-Associated Archaeome.”; still, we observed a total of 39 *Methanothermobacter* ASVs spanning 78 samples (mean of 18 ± 30 s.d. samples per ASV), which strongly suggests that its presence is not due to contamination. Moreover, the top BLASTn hit for 36 of the 39 ASVs was to a cultured *Methanothermobacter* strain (Figure S23, Table S6), including the top 15 most abundant ASVs, which indicates that the taxonomic annotations are demonstrably correct.

The high prevalence of *Methanothermobacter* among *Aves* lead us to the hypothesis that body temperature significantly affects the distribution *Methanothermobacter* (Figure S24), given that birds generally have higher body temperatures than mammals Clarke and O’Connor, “Diet and Body Temperature in Mammals and Birds.” and all existing *Methanothermobacter*

cultures are thermophiles Bonin and Boone, “The Order Methanobacteriales.”. Moreover, *Methanothermobacter* is not abundant in *Monotremata* and *Marsupialia* species relative to the placental groups, which reflects a lower body temperature in the latter clades (Figure S24). We were able to assign published body temperature data to 73 mammalian and avian species (Figure S25A & S25B; Table S7). Genus-level abundances of *Methanothermobacter* significantly correlated with body temperature (RRPP, adj. $P < 0.001$), while *Methanobrevibacter* and *Methanosphaera* did not (Figures S25C & S25D). However, the association was only significant if not accounting for host phylogeny (RRPP, adj. $P > 0.05$), indicating that the association between *Methanothermobacter* and body temperature could not be decoupled from host evolutionary history. We also identified 7 *Methanothermobacter* ASVs to be correlated with body temperature (RRPP, adj. $P < 0.05$; Figure S25E), while no *Methanobrevibacter* or *Methanosphaera* ASVs were correlated. Again, the association was only significant if not accounting for host phylogeny. Regardless, we provide evidence congruent with the hypothesis that *Methanothermobacter* abundance is modulated by host body temperature and is thus rather highly abundant in birds and various placental mammal clades.

qPCR with novel *Methanothermobacter*-targeting primers supported our findings that *Methanothermobacter* is present among many avian and mammalian species (Figure S27). Notably, *Methanothermobacter* presence/absence was consistent for members of the same host species. *Methanothermobacter* gene copies per gram of GI sample varied from approximately 5e3 - 5e6. The qPCR data did not show as widespread of *Methanothermobacter* prevalence as was seen for the 16S rRNA sequence data (Figure S24), but this may be due to the low amount of animal GI sample gDNA available for the assay, or possibly due to primer biases limiting the amount of *Methanothermobacter* diversity detected by the qPCR primers (*i.e.,* the primers we designed from existing genomes, which may be rather divergent from host-associated *Methanothermobacter*). Due the the smaller sample size of the qPCR assay relative to the 16S rRNA sequence data, and the lack of overlapping host body temperature data with the qPCR findings, we could not use these additional data to support or refute the hypothesis that host body temperature modulates *Methanothermobacter* abundance (Figure S27). Still, the 16S rRNA sequence data provides compelling evidence that host body temperature modulates *Methanothermobacter* abundance (Figures S24 & S25).

Metagenome assembly of avian samples also supported our findings of high *Methanothermobacter* prevalence across *Aves*. We mapped all assembled contigs (≥1.5 kb) to species representative genomes of the *Methanothermobaceteriales* (GTDB Release 95 taxonomy). The number of contigs that mapped to *Methanothermobacteraceae* or *Methanothermobacteraceae A* was 88 ± 799 (s.d.), which was 0.88% ± 0.93 (s.d.) of all contigs assembled. Still, we were not able to assemble a genome classified as the target clade, possibly due to high intra-sample strain diversity, as suggested by the ASV data (Table S6).

We note that among the host species in which methane emission data exists Hackstein and van Alen, “Fecal Methanogens and Vertebrate Evolution”; Clauss et al., “Review: Comparative Methane Production in Mammalian Herbivores.”, avian species with high abundances of *Methanothermobacter* have emission rates on the higher end of mammal emission rates (Figure S29), suggesting that *Methanothermobacter* is indeed a persistent inhabitant in the gut of some avian species.

Microbe-microbe interactions modulating archaeal diversity

Besides host-specific factors potentially modulating diversity, microbe-microbe interactions may also play a significant role. We first tested for solely archaeal interactions by inferring instances of co-occurrence among archaeal ASVs. The co-occurrence network contained clearly defined subnetworks, with few significant positive associations between them (Figure S31), especially for the largest 6 subnetworks (Figure S30). The only significant negative co-occurrences were between Subnetwork 1, which was dominated by *Methanobrevibacter*, and Subnetwork 4, which was dominated by *Methanothermobacter*. These 2 subnetworks differed substantially in their distributions across host clades, with Subnetwork 1 ASVs only highly prevalent among *Artiodactyla*, while Subnetwork 4 ASVs were highly prevalent across a number of mammalian orders (*e.g., Carnivora* and *Rodentia*) and almost all avian orders (Figure S32). Among subnetworks, ASV taxonomy was highly homogeneous. Indeed, we found ASVs to significantly and strongly associated with those of the same clade versus from other clades, regardless of taxonomic level (Figure S30C), although assortativity by taxonomic affiliation substantially dropped between the family and genus levels.

We investigated potential diet-specific archaea-archaea interactions by separately testing for co-occurrences across samples of each diet (Figure S33). The number of significant co-occurrences dropped from herbivores (n = 560) to omnivores (n = 134) to carnivores (n = 81). In contrast, assortativity by taxonomic group was generally lowest for omnivores and highest for carnivores, regardless of taxonomic level. These findings suggest that the carnivore gut is composed of simpler and more taxonomically homogenous archaeal consortia relative to omnivores and herbivores.

We also assessed *Bacteria-Archaea* interactions by utilizing the overlapping 16S-uni dataset samples from Youngblut and colleagues Youngblut et al., “Host Diet and Evolutionary History Explain Different Aspects of Gut Microbiome Diversity among Vertebrate Clades.”. Prior to merging the datasets, we removed all archaeal ASVs from the 16S-uni dataset. Archaeal and bacterial alpha diversity were not correlated, regardless of measuring diversity via the Shannon Index or Faith’s PD (Pearson, P > 0.05; Figure 4). Moreover, archaeal and bacterial beta diversity were not correlated (Mantel, P > 0.05; Procrustes superimposition, P > 0.05), regardless of the measure: Bray-Curtis, Jaccard, and weighted/unweighted UniFrac. These results suggest that archaeal diversity is not explained by bacterial diversity nor vice versa.

Inferring a co-occurrence network of bacterial and archaeal ASVs revealed a large number of significant co-occurrences (n = 3018; Figure 4); all of which were positive. *Bacteria*-*Archaea* and *Archaea*-*Archaea* associations comprised 13.1 and 6.1% of the network edges, respectively. While overall network taxonomic assortativity was low, assortativity of just *Archaea* was quite high (≥0.774 for all taxonomic levels). The entire network comprised 5 subnetworks, but only 2 included *Archaea*: one of which included only *Methanobrevibacter* ASVs, while the other was dominated by *Methanothermobacter* ASVs. The *Methanobrevibacter*-only subnetwork also comprised 13 bacterial families from 3 phyla. *Firmicutes* dominated among the bacterial ASVs (87%), with *Bacteroidetes* as a distant second (11%). The most represented bacterial families in the network were *Ruminococcaceae* (46%), *Lachnospiraceae* (13%), and *Christensenellaceae* (11%), which are known include hydrogen generating species that often occur with *Methanobrevibacter* Hansen et al., “Pan-Genome of the Dominant Human Gut-Associated Archaeon, Methanobrevibacter Smithii, Studied in Twins”;

Goodrich et al., “Human Genetics Shape the Gut Microbiome”; Borrel et al., “The Host-Associated Archaeome.”. The *Methanothermobacter*-dominated subnetwork included much less bacterial diversity, with only 3 families: *Burkholderiaceae* (*Proteobacteria* phylum); *Enterococcaceae* and *Clostridiaceae 1* (*Firmicutes* phylum). These findings indicate that a subset of archaeal ASVs co-occur with specific bacterial ASVs in each of the 2 consortia: the *Methanothermobacter*-dominanted consortium most prevalent among birds and the *Methanobrevibacter*-dominated consortium most prevalent among ruminants and various other plant-consuming mammals (Figure S24). While only methanogens were observed to co-occur with bacteria, this may be due to the mammalian bias of the dataset, given that prevalence of non-methanogenic archaea is lower among mammals relative to other vertebrate classes (Figure 1).

Supplemental Tables

Table S1. All relevant metadata for all samples in the 16S rRNA amplicon dataset.

Table S2. Metadata for all samples in which *Archaea*-targeted 16S rRNA amplicon library preparation and sequencing was attempted (*n* = 311) and the samples that passed all quality control measures (*n* = 185). "mass stored (g)" is the initial mass (g) of the gastrointestinal sample. "Nanodrop (ng/ul)" is the Nanodrop quantification of gDNA concentration (ng/ul). "Nanodrop 260:280" is the Nanodrop quantification of gDNA 260:280 ratios. "16S rRNA gene copies per 100 ul" is the estimated microbial 16S rRNA gene copy number based on qPCR with "universal" primers (see Methods).

Table S3. The total archaeal sequence counts per sample (*n* = 185).

Table S4. Percent relative abundance of each archaeal taxonomic class in each sample (*n* = 185). Classes are labeled as "Phylum;Class".

Table S5. Genus-level percent relative abundances of *Bathyarchaeia* in all samples where the clade was detected.

Table S6. The top 5 BLASTn hits of all *Methanothermobacter* ASV sequences to the All Species Living Tree v132 database (see Methods). Mean percent relative abundances across all samples and samples grouped by host taxonomic class are also provided.

Table S7. Publicly available body temperature data used in this study. If multiple temperature data points per species were available, the mean temperature was used. The datasets include "Clarke2010" Clarke, Rothery, and Isaac, "Scaling of Basal Metabolic Rate with Body Mass and Temperature in Mammals.", "Clarke2014" Clarke and O'Connor, "Diet and Body Temperature in Mammals and Birds.", "McNab1966" McNab, "The Metabolism of Fossorial Rodents: A Study of Convergence.", "Prinzinger1991" Prinzinger, Preßmar, and Schleucher, "Body Temperature in Birds.", "Riek2013" Riek and Geiser, "Allometry of Thermal Variables in Mammals: Consequences of Body Size and Phylogeny.", "Sieg2009" Sieg et al., "Mammalian Metabolic Allometry: Do Intraspecific Variation, Phylogeny, and Regression Models Matter?", and "Teare2002" Teare, "International Species Information System, Medical Animal Records Keeping System (MedARKS) 2002 Data Extraction.". "No match" indicates the species lacking a match to any of the body temperature datasets; these species were not included in any analyses of body temperature due to a lack of data.

Table S8. Publicly available animal methane emission data used in this study. The studies comprise "Hackstein_1996" Hackstein and van Alen, "Fecal Methanogens and Vertebrate Evolution." and "Clauss_2020" Clauss et al., "Review: Comparative Methane Production in Mammalian Herbivores.".

Table S9. All significant *Archaea*-*Bacteria* ASV co-occurrences, summarized by genus and direction (*i.e.,* positive or negative) and normalized by the total number of edges in the co-occurrence network. “Sign” indicates whether the association was positive or negative.

Table S10. qPCR data with “universal” 16S rRNA primers (see Methods). “mass_stored_g” is the amount of GI sample used for DNA extractions. “Copies_per_100_ul” is the qPCR-estimated 16S rRNA copies per 100 µl of gDNA.

Table S11. NCBI accessions of publicly available *Methanothermobacter* genomes used for *Methanothermobacter*-targeted primer design.

Table S12. All *Methanobacteriales* species-level reference genomes used for metagenome assembly contig mapping. Reference genomes were selected based on highest CheckM-estimated completeness and lowest CheckM-estimated contamination.

Supplemental Figures

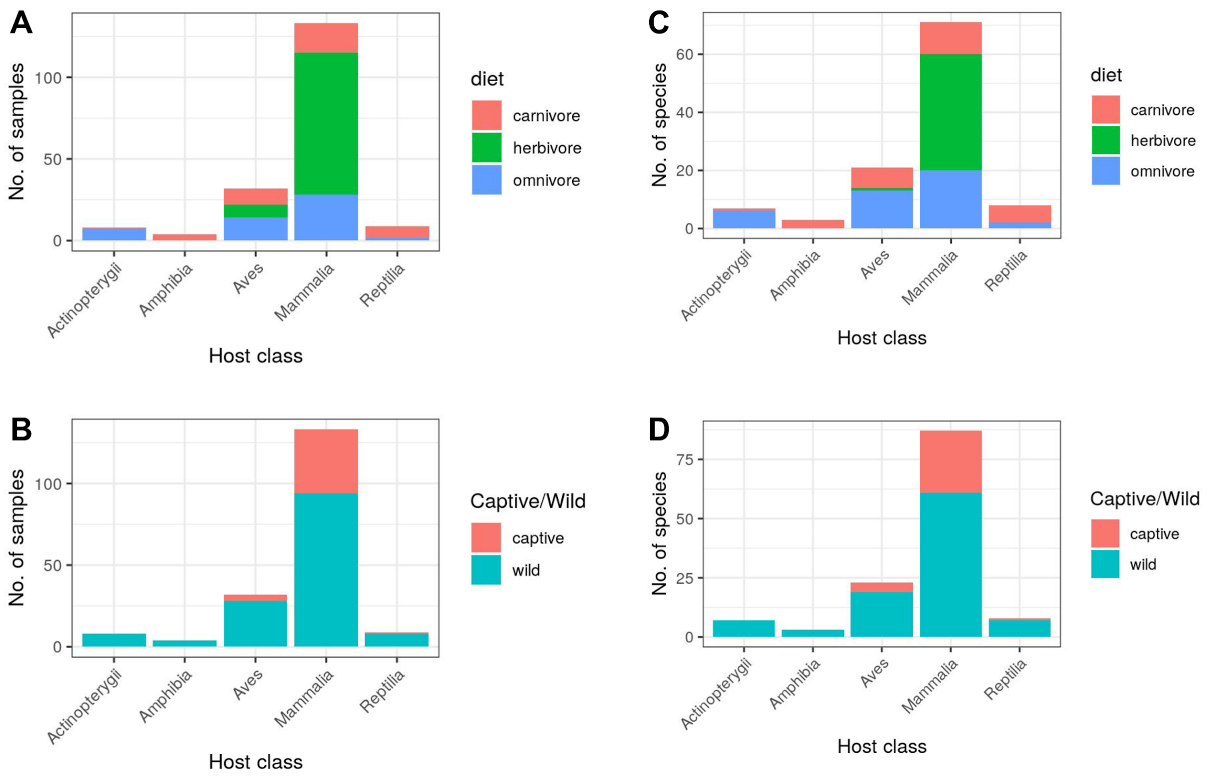

Figure S1. The number of samples (A & B) or host species (C & D) in the final sequence dataset, grouped by host class, host diet (A & C) or host captive/wild status (B & D).

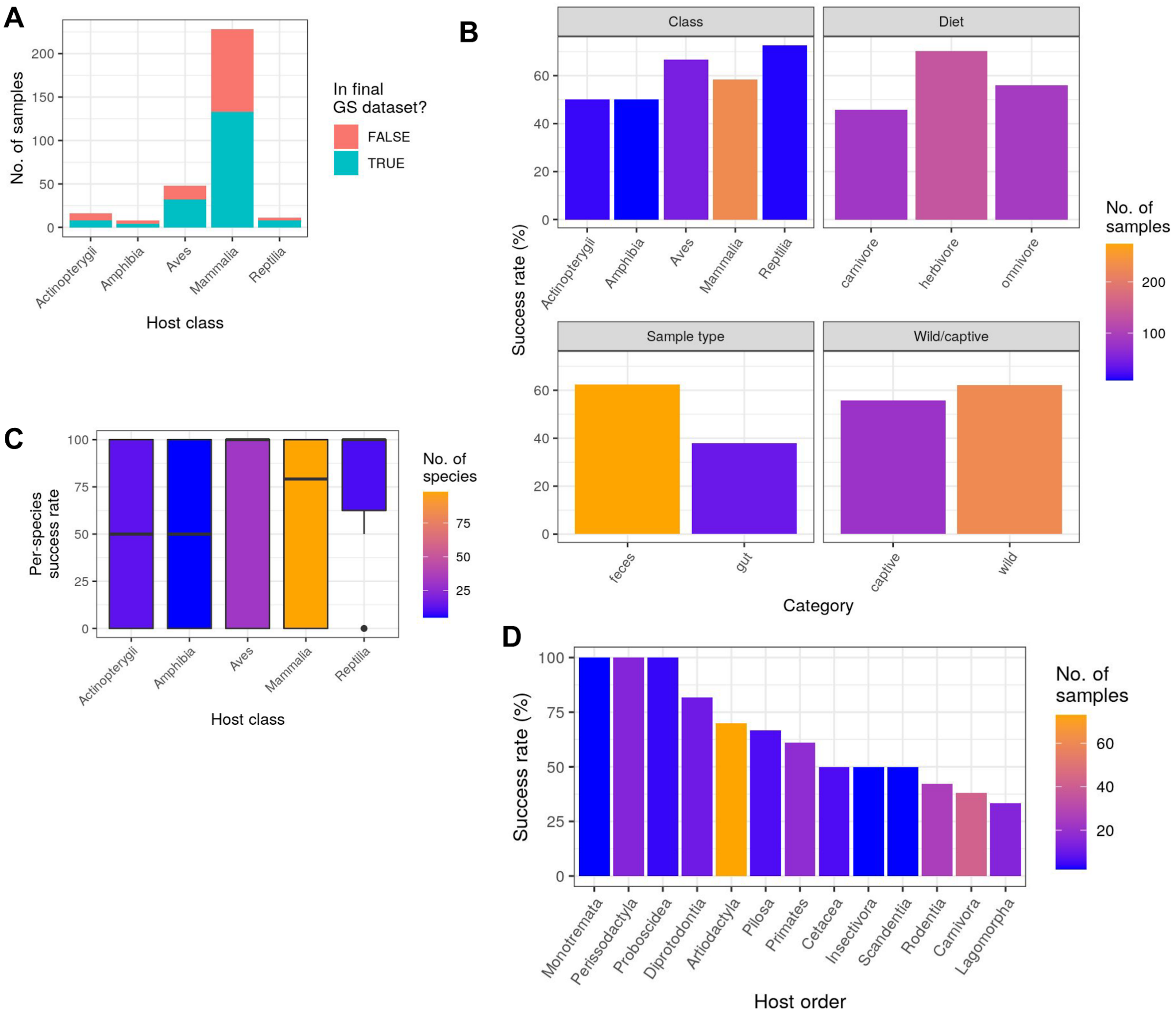

Figure S2. A) The number of samples that passed or failed PCR amplification and sequence data quality control. B) The percent of total samples that passed PCR amplification and sequence data quality control (*i.e.,* the success rate), with values grouped by various host metadata categories. C) The success rate among individuals of the same species, grouped by host class. D) The success rate for each mammalian taxonomic order. See Table S2 for a list of all successes and failures.

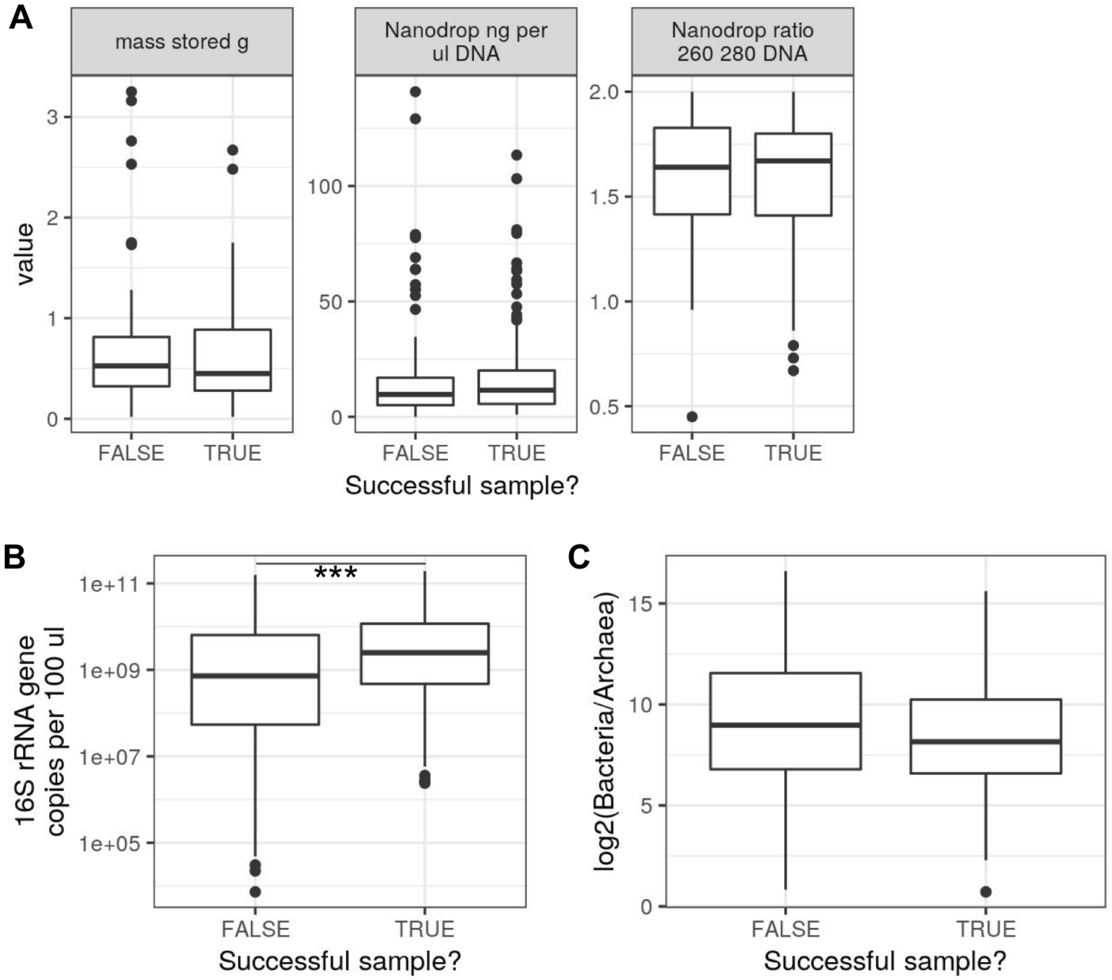

Figure S3. *qPCR estimates of 16S rRNA gene copies (16S "universal" primers) significantly differ between samples that passed 16S-arc PCR amplification and sequencing versus those that failed.* "Successful sample" refers to whether the sample passed 16S-arc PCR amplification and sequence data quality control. "mass stored g" is the initial mass (g) of the gastrointestinal sample. "Nanodrop ng per ul DNA" is the Nanodrop quantification of gDNA concentration (ng/ul). "Nanodrop ratio 260 280 DNA" is the Nanodrop quantification of gDNA 260:280 ratios. "16S rRNA gene copies per 100 ul" is the estimated microbial 16S rRNA gene copy number based on qPCR with "universal" primers (see Methods and Table S2). "log2(Bacteria/Archaea)" is the $\log_2$-fold ratio of bacterial relative abundance versus archaeal relative abundance, as estimated by utilizing metagenome reads from animal gut sample in Youngblut and colleagues Youngblut et al., "Large-Scale Metagenome Assembly Reveals Novel Animal-Associated Microbial Genomes, Biosynthetic Gene Clusters, and Other Genetic Diversity." that overlapped with the samples used in this study (n = 258). Taxon abundances were estimated from the metagenome reads via Kraken2 and Bracken with a custom reference database generated from the GTDB (Release 95) (see Methods). Box centerlines, edges, whiskers, and points signify the median, interquartile range (IQR), 1.5 × IQR, and >1.5 × IQR, respectively. See the Statistical_source_data file for all statistical information.

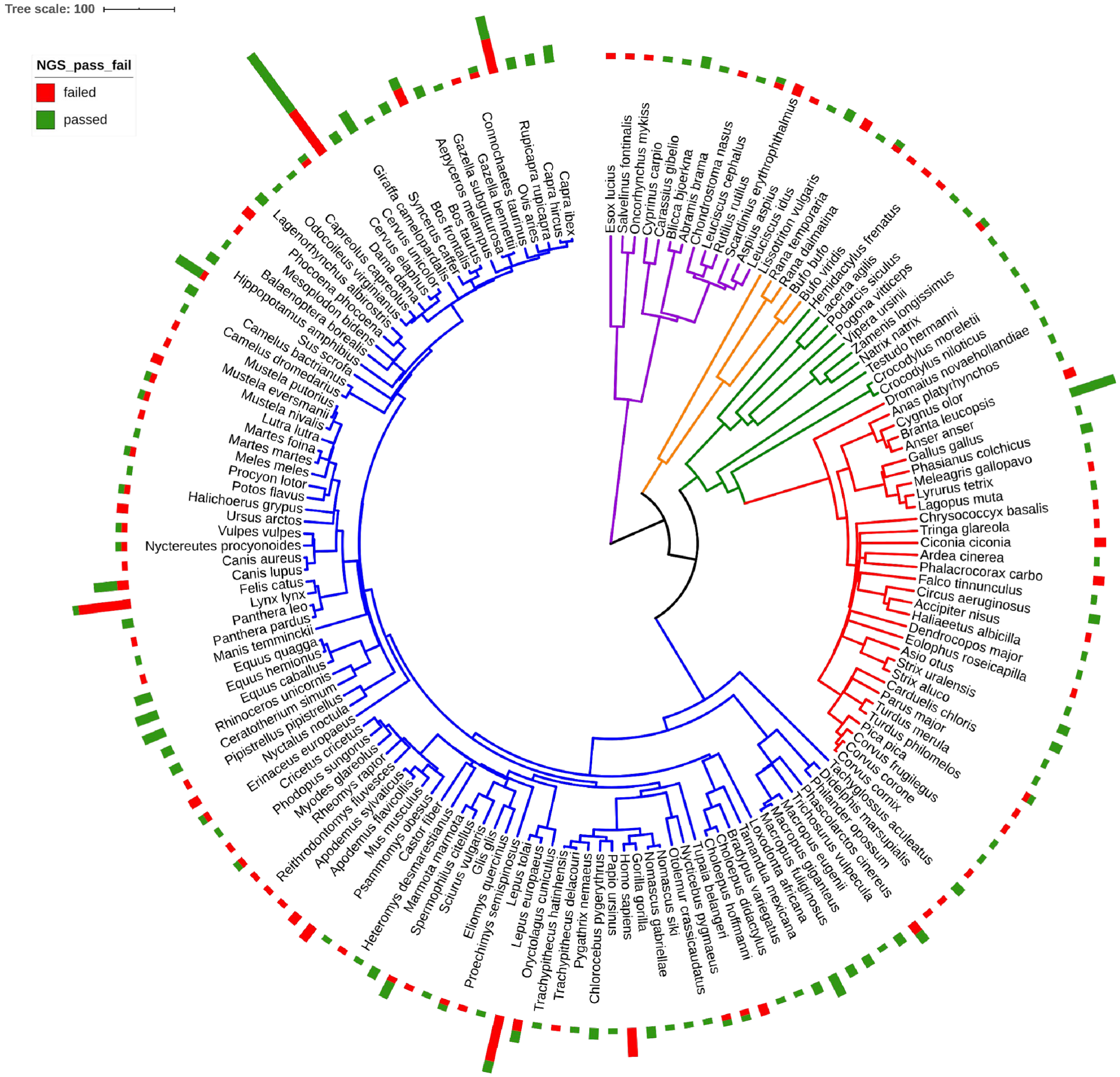

Figure S4. The number of samples that passed PCR amplification and sequence data quality control (“passed”) and those that failed (“failed”) mapped onto a phylogeny of all host species. The phylogeny is the same as shown in Figure 1.

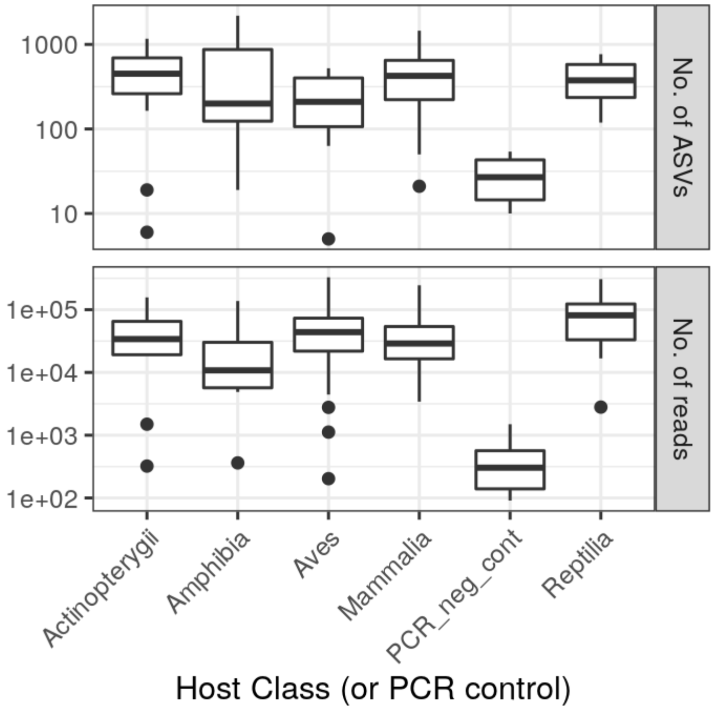

Figure S5. The number of raw ASVs per sample, grouped by host class and ASV abundances prior to filtering out Bacteria and rarefying. "PCR_neg_cont" denotes all PCR negative controls that were sequenced along with the treatment samples (*n* = 10). Note that the y-axis is $\log_{10}$-transformed, and note that some of the treatment samples shown were filtered during rarefying to ≥250 sequences. Only 6 genera had a mean percent abundance of ≥10% in the negative controls: *Catellicoccus* (*Firmicutes*), *Catenibacterium* (*Erysipelotrichia*), *Lactobacillus* (*Bacilli*), *Bacillus* (*Bacilli*), *Lactococcus* (*Firmicutes*), *Ruminococcaceae UCG-014* (*Firmicutes*). Box centerlines, edges, whiskers, and points signify the median, interquartile range (IQR), 1.5 × IQR, and >1.5 × IQR, respectively.

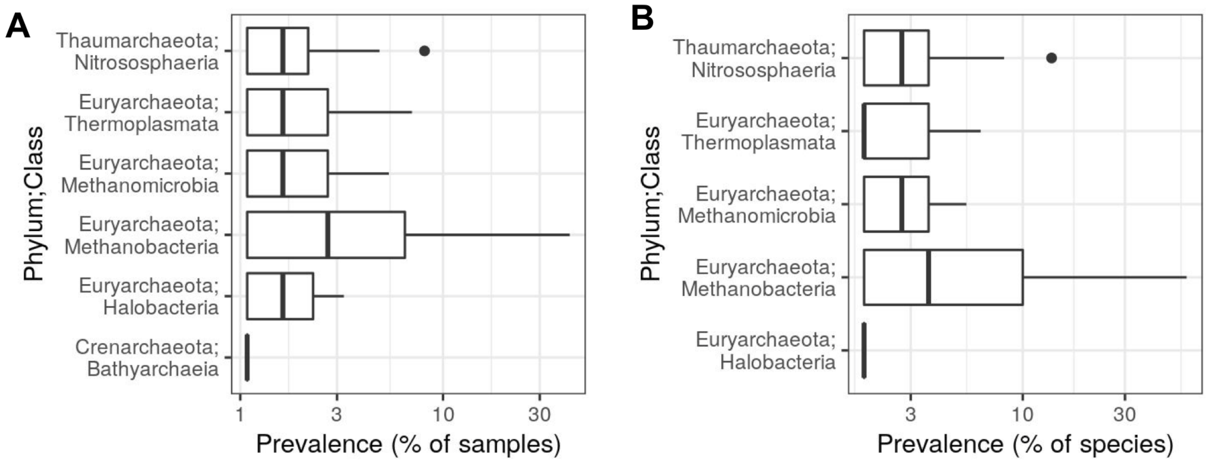

Figure S6. The prevalence of archaeal ASVs detected in A) >1 sample (*n* = 377) or B) >1 species (*n* = 315). Box centerlines, edges, whiskers, and points signify the median, interquartile range (IQR), 1.5 × IQR, and >1.5 × IQR, respectively.

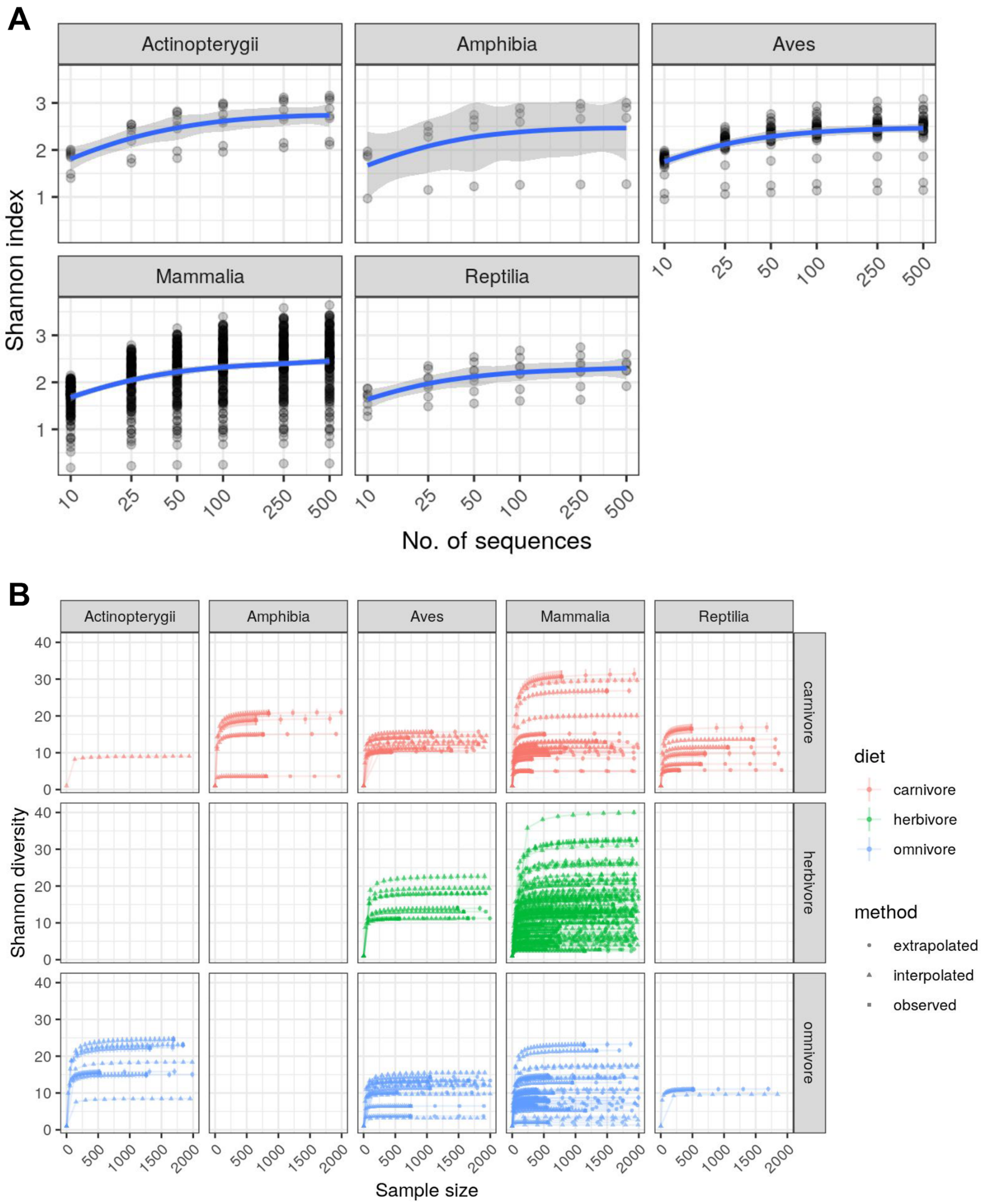

Figure S7. A) Rarefaction grouped by host taxonomic class, with subsampling continued up to 500 per sample (if possible, depending on the sample). The blue lines are a smoothed curve fit, with grey regions denoting the 95% CI. B) Rarefaction with extrapolation via iNEXT, with subsampling/extrapolation up to 2000 per sample. Diversity was measured as Hill numbers (diversity order of 1, which is equivalent to Shannon diversity). Error bars denote the 95% CI.

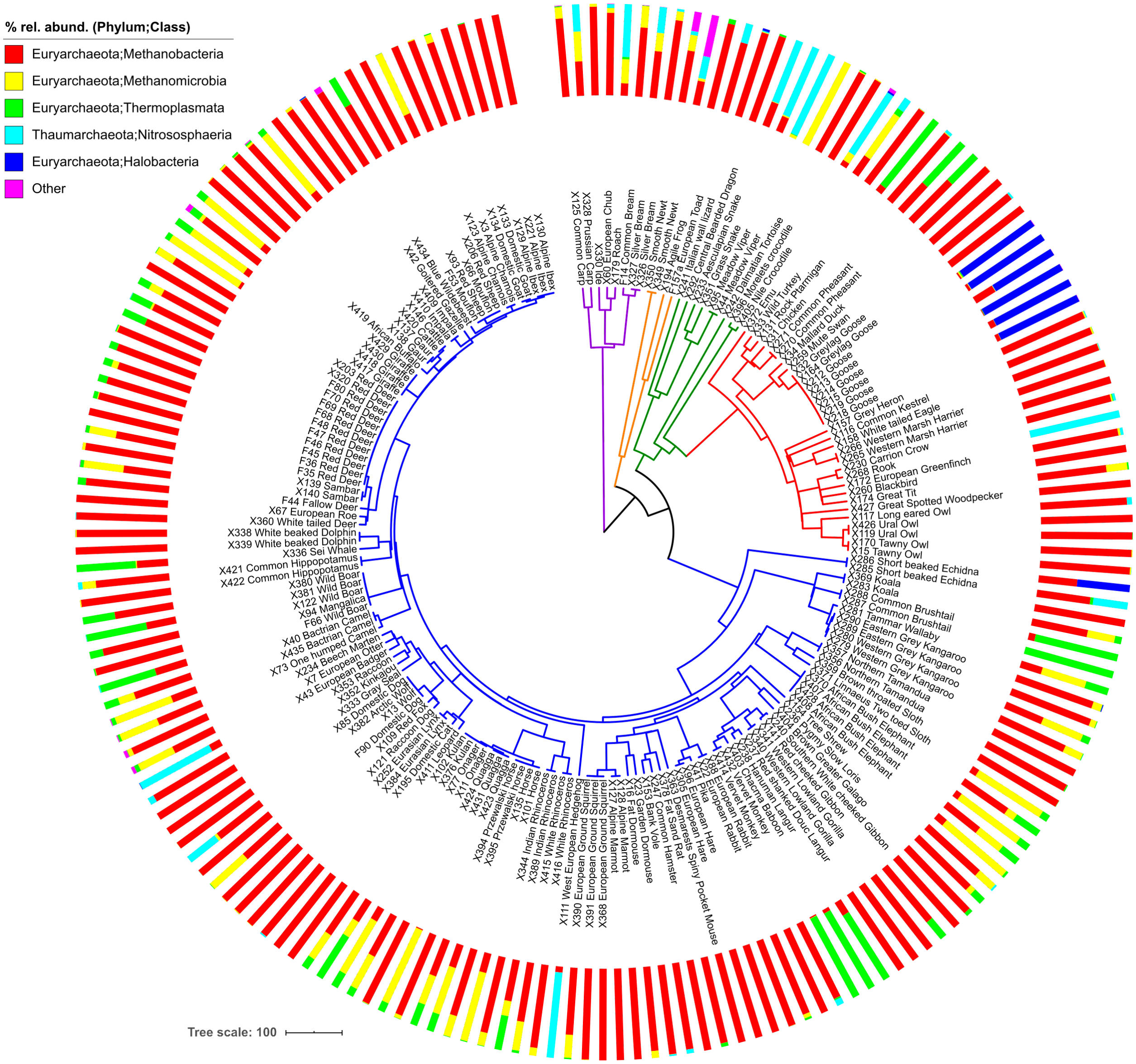

Figure S8. The host phylogeny is the same as shown in Figure 1, except tips have been expanded to include all individuals of each species (n = 185). Relative abundances of ASVs aggregated by taxonomic class are mapped onto the tree. All classes with <1% mean abundance are labeled as “Other”, which includes *Woesearchaeia*, *Thermococci*, *Iainarchaeia*, and *Odinarchaeaia*.

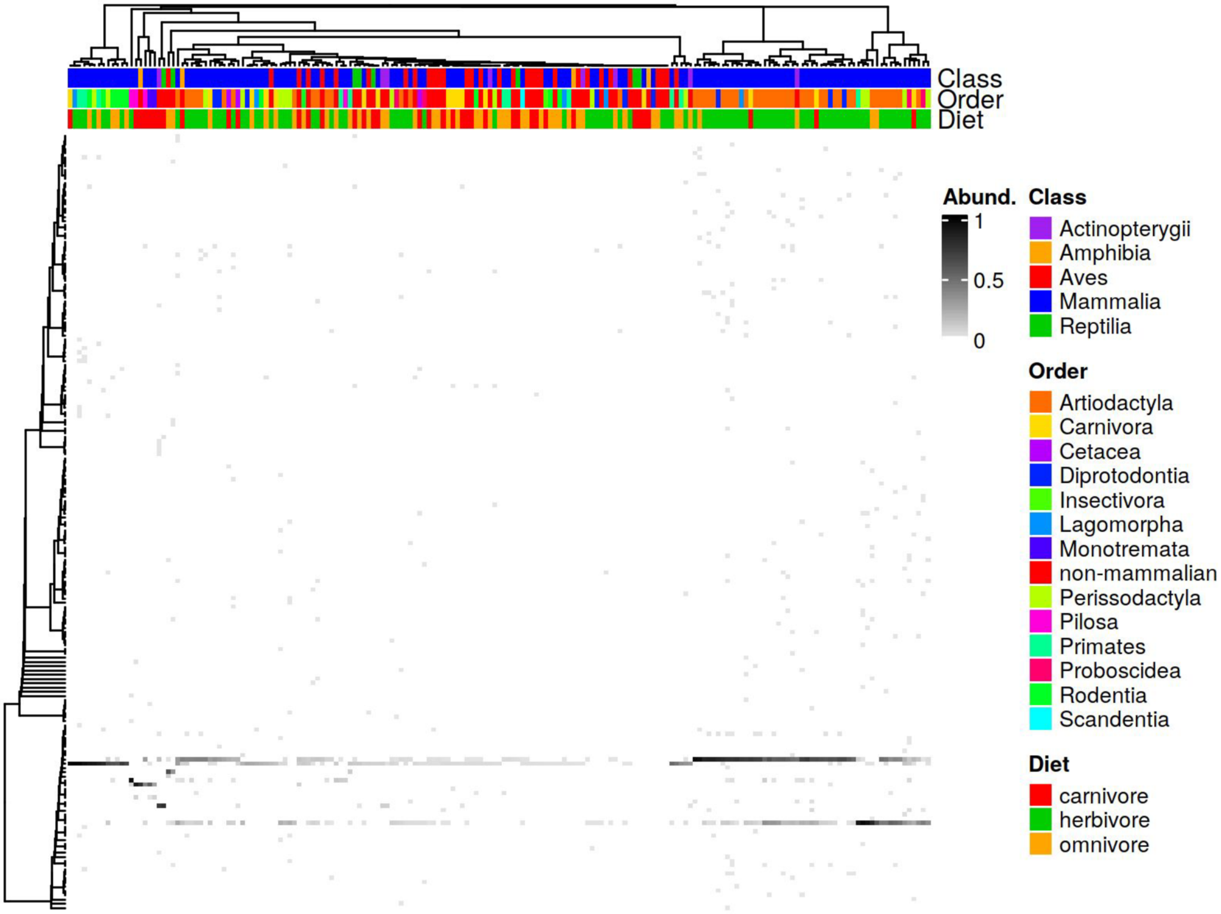

Figure S9. Methanobrevibacter *is dominated by a few strains with varying abundance distributions.* The heatmap shows fractional abundances for all *Methanobrevibacter* ASVs, collapsed at 0.05 patristic distance to reduce the number of taxa from 699 ASVs to 183 representatives. The abundances are percentages of all archaeal taxa in the sample. The tree on the left is the 16S rRNA sequence phylogeny of all archaeal ASVs pruned to the 183 representatives. The pruned phylogeny was forced to ultrametric for visualization. Hierarchical clustering of all samples (*n* = 185) is shown via the dendrogram above the heatmap. For clarity, only mammalian taxonomic orders are shown.

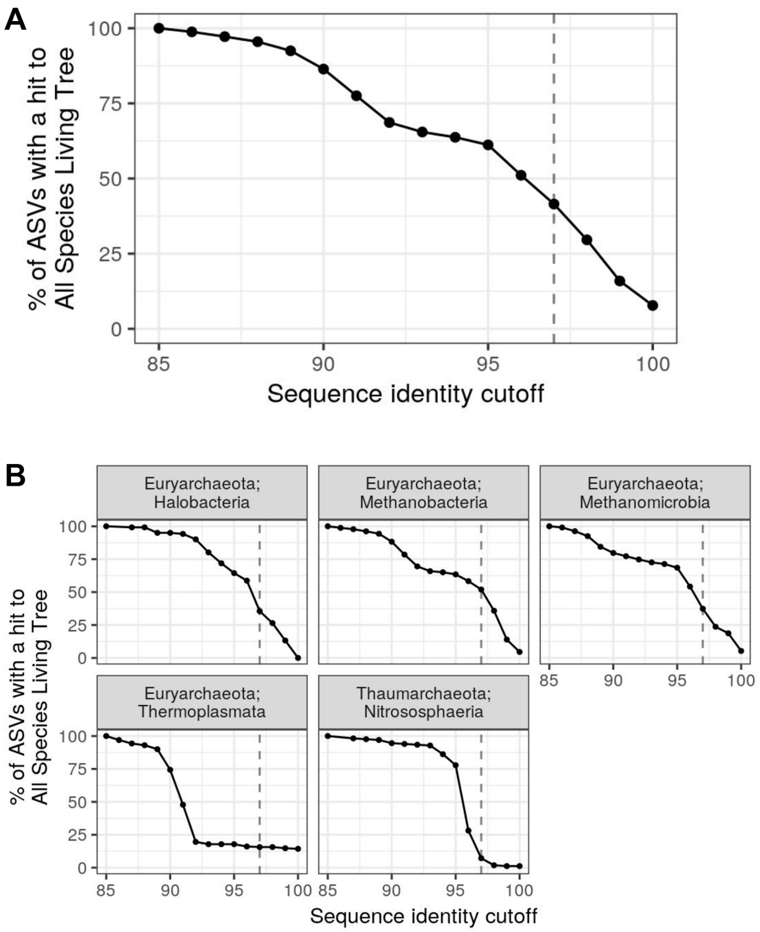

Figure S10. *Substantial uncultured archaeal diversity even among relatively well-studied clades*. The percent of ASVs with a ≥1 BLASTn hit to a culture representative in the All Species Living Tree database v132 (hit alignment length ≥95% of the query), depending on the sequence identity cutoff of the BLASTn hit. Values are shown for A) all ASVs and B) ASVs grouped by taxonomic class (facet labels are “Phylum; Class”) for the subset of classes in which any hits were observed along the range of sequence identity cutoffs shown.

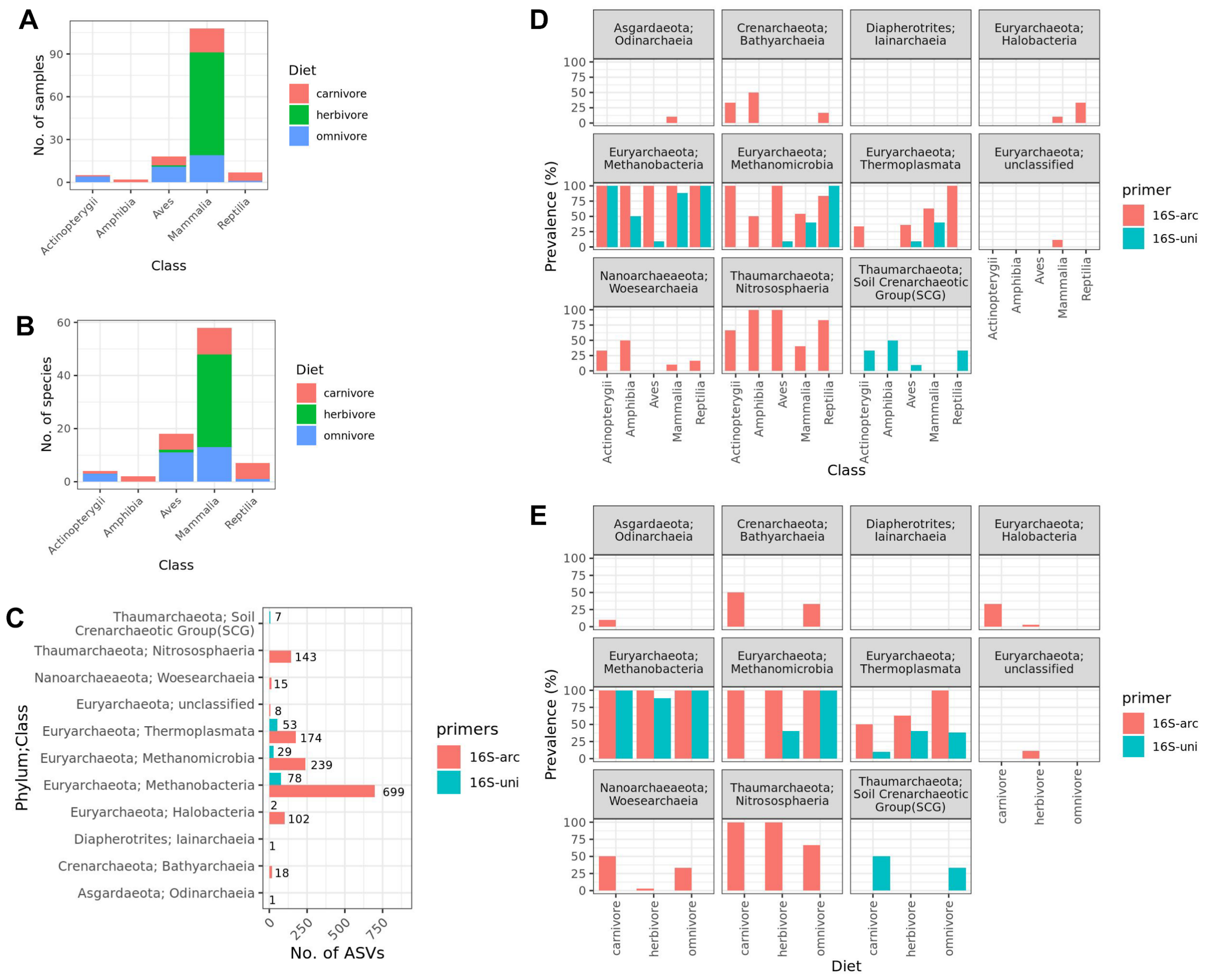

Figure S11. *The* Archaea-*targeting primer set revealed much more archaeal diversity than standard "universal" 16S rRNA NGS primers.* The number of A) samples or B) host species that overlap between the 16S-arc and 16S-uni amplicon sequence datasets. C) The number of archaeal ASVs per sequence dataset. D) & E) The number of archaeal classes across host species grouped by D) host taxonomic class or E) diet. A total of 140 samples overlapped between the two datasets. ASVs are specific to each primer set; we are just comparing the number of archaeal ASVs generated by each primer set and did not try to match ASV sequences between sequence datasets.

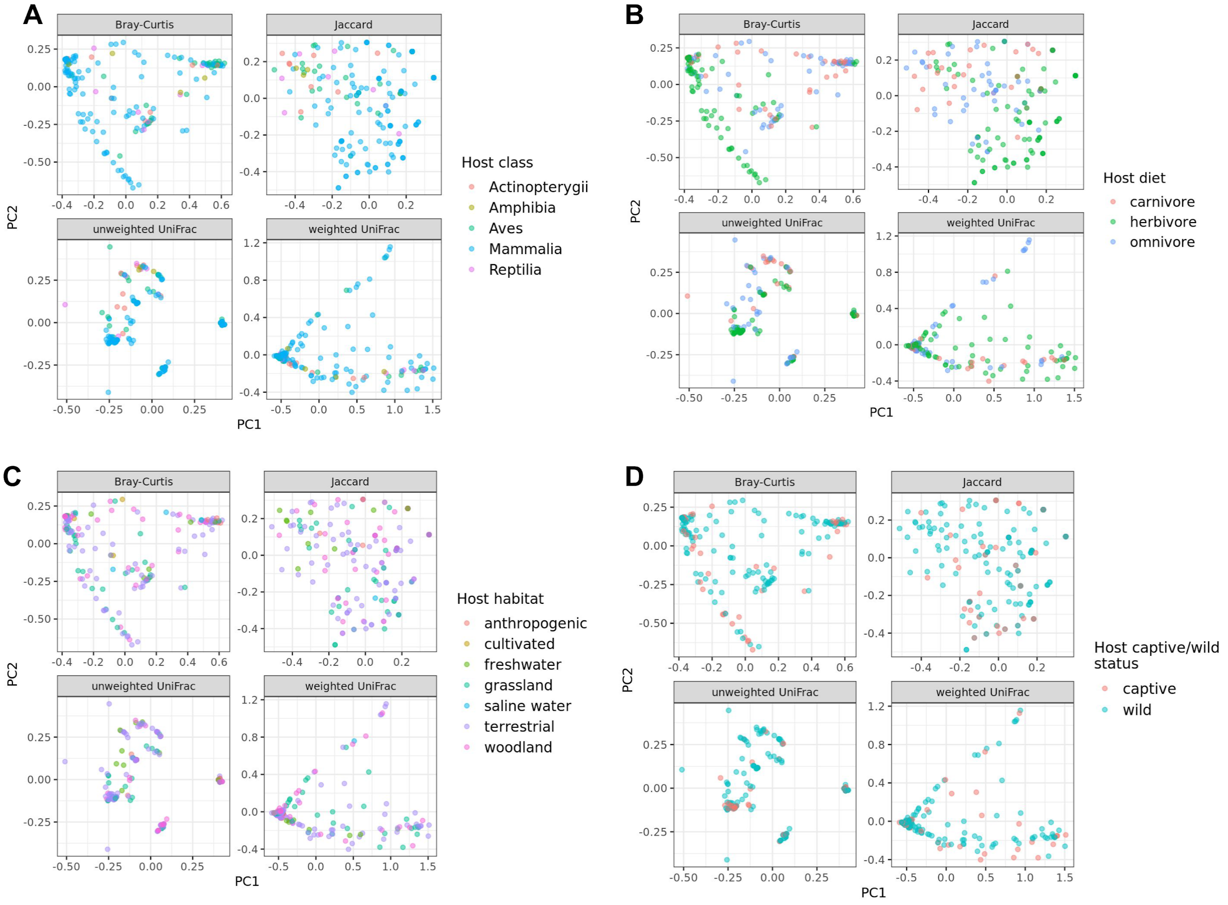

Figure S12. *Principal coordinates plots qualitatively agree with the MRM analysis results*. Principal coordinates (PCoA) ordinations of unweighted and weighted Unifrac, Jaccard, and Bray-Curtis distances among all samples, with samples colored by host A) class, B) diet, C) habitat, and D) captive/wild status. The percent variance explained by PC1 and PC2 is 18 & 9% for Bray-Curtis, 14 and 6% for Jaccard, 29 and 19% for unweighted UniFrac, and 72 and 12% for weighted UniFrac, respectively.

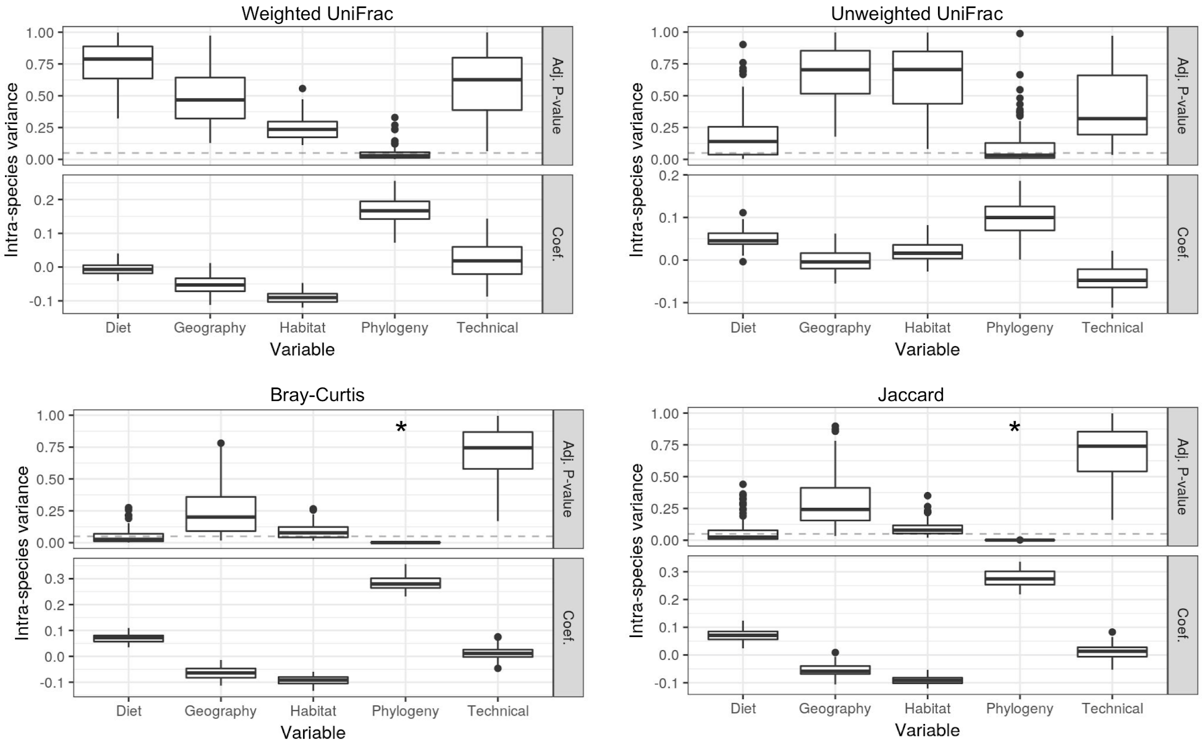

Figure S13. *Host phylogeny shows the strongest association to archaeal community beta diversity for mammalian species.* The plots show the distribution of *P*-values ("Adj. P-value") and partial regression coefficients ("Coef.") across 100 dataset permutations used for multiple regression on matrix (MRM) tests. Unlike Figure 2A, only mammalian species were included, leaving 71 mammalian species. For each permutation, one individual per host species was randomly sampled. MRM tests assessed the beta diversity variance explained by host diet, geography, habitat, phylogeny, and "technical" parameters (see Methods), with 4 beta diversity measures assessed: A) weighted UniFrac, B) unweighted UniFrac, C) Bray-Curtis, and D) Jaccard. Asterisks denote significance (adj. $P < 0.05$ for >95% of dataset subsets; see Methods). Beta diversity calculated on ASVs aggregated at the genus level. Box centerlines, edges, whiskers, and points signify the median, interquartile range (IQR), 1.5 × IQR, and >1.5 × IQR, respectively. The MRM tests were two-sided. See the Statistical_source_data file for all other statistical information.

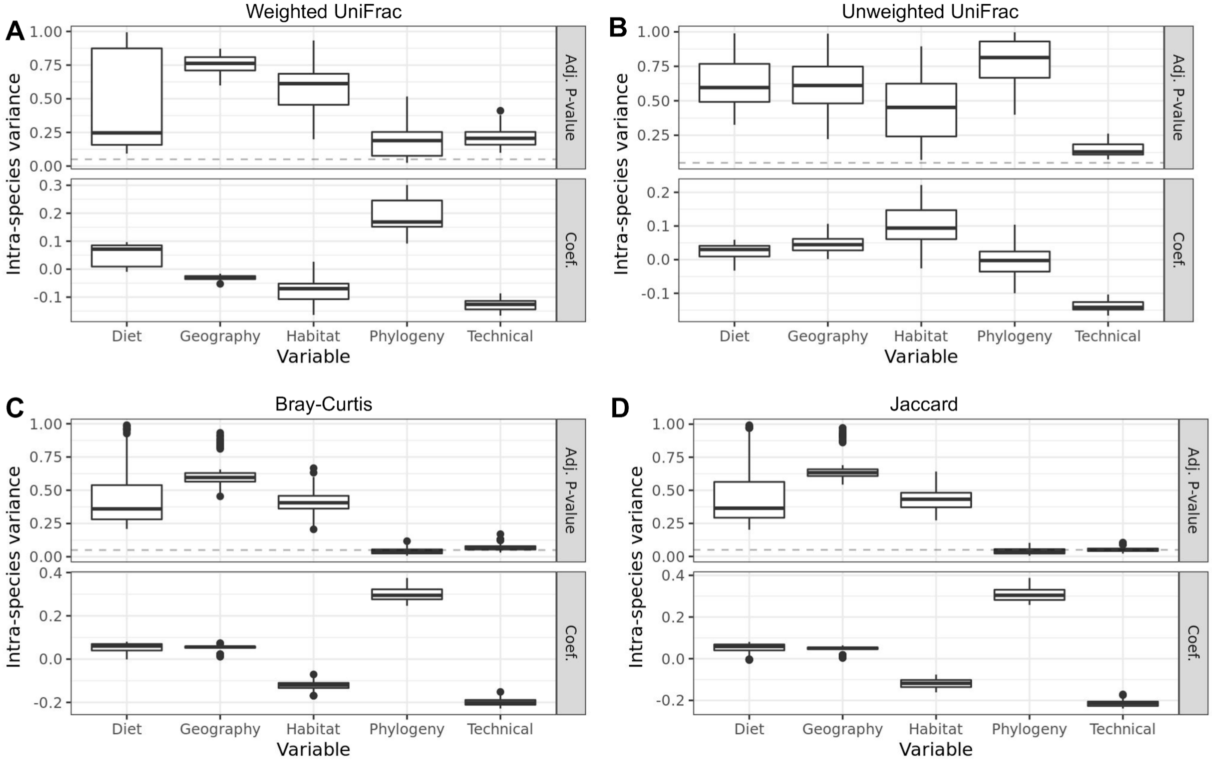

Figure S14. *Host phylogeny trending to significance for non-mammalian species.* The plots show the distribution of *P*-values ("Adj. P-value") and partial regression coefficients ("Coef.") across 100 dataset permutations used for multiple regression on matrix (MRM) tests. Unlike Figure 2A, all mammalian species were excluded, leaving 39 non-mammalian species. For each permutation, one individual per host species was randomly sampled. MRM tests assessed the beta diversity variance explained by host diet, geography, habitat, phylogeny, and "technical" parameters (see Methods), with 4 beta diversity measures assessed: A) weighted UniFrac, B) unweighted UniFrac, C) Bray-Curtis, and D) Jaccard. Asterisks denote significance (adj. $P < 0.05$ for >95% of dataset subsets; see Methods). Beta diversity calculated on ASVs aggregated at the genus level. Box centerlines, edges, whiskers, and points signify the median, interquartile range (IQR), 1.5 × IQR, and >1.5 × IQR, respectively. The MRM tests were two-sided. See the Statistical_source_data file for all other statistical information.

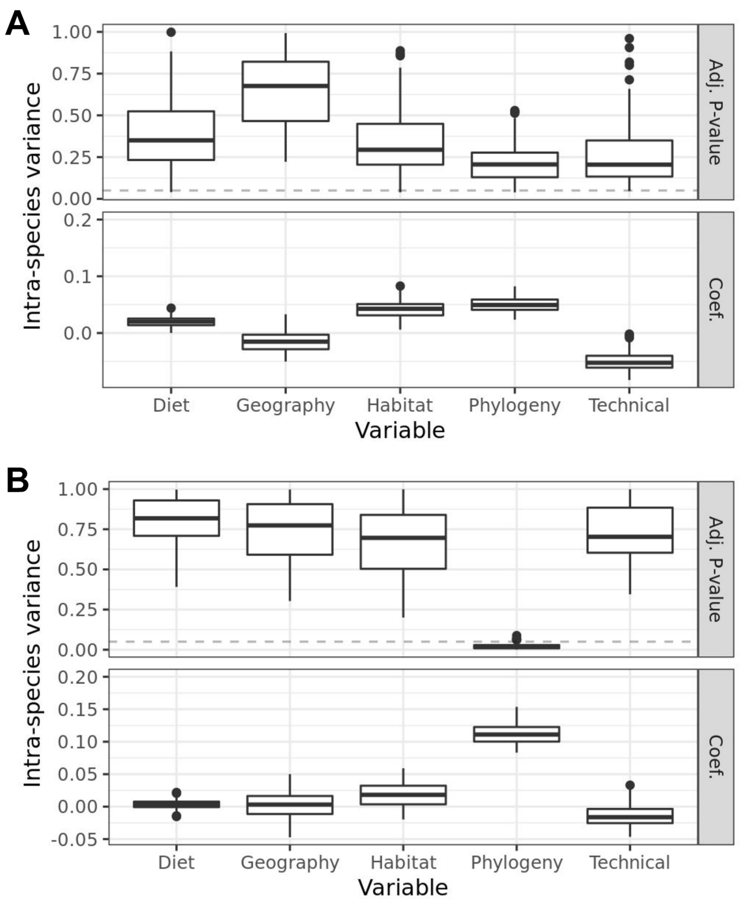

Figure S15. *No host factors significantly explain archaeal alpha diversity*. The plots show the distribution of *P*-values ("Adj. P-value") and partial regression coefficients ("Coef.") across 100 dataset permutations used for multiple regression on matrix (MRM) tests. For each permutation, one individual per host species was randomly sampled. MRM tested whether inter-sample variance of alpha diversity was significant explained by host diet, geography, habitat, phylogeny, and "technical" parameters (see Methods), with 2 alpha diversity measures assessed: A) Shannon Index and B) Faith's PD. No variables were significant (defined as adj. $P < 0.05$ for >95% of dataset permutations; see Methods). Box centerlines, edges, whiskers, and points signify the median, interquartile range (IQR), 1.5 × IQR, and >1.5 × IQR, respectively. The MRM tests were two-sided. See the Statistical_source_data file for all other statistical information.

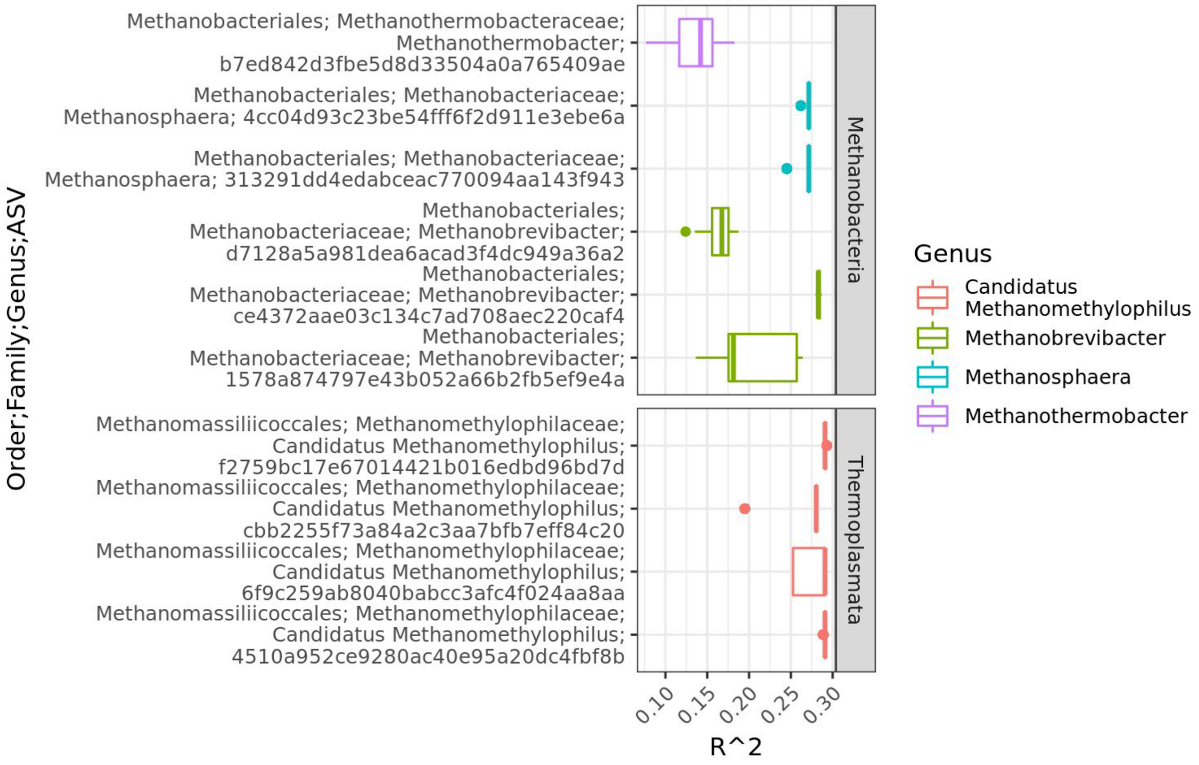

Figure S16. *Certain methanogen ASVs from multiple lineages are associated with diet, after accounting for host phylogeny*. Phylogenetic generalized least squares (PGLS) results for the ASVs with a significant association between ASV abundance and host diet, while accounting for host phylogenetic relatedness. Significance was defined as adj. $P < 0.05$ in ≥95% of permuted datasets, in which one sample per species was used per permutation. The boxplots depict the distribution of PGLS R^2 values across all 100 permutations. Box centerlines, edges, whiskers, and points signify the median, interquartile range (IQR), 1.5 × IQR, and >1.5 × IQR, respectively. See the Statistical_source_data file for all other statistical information.

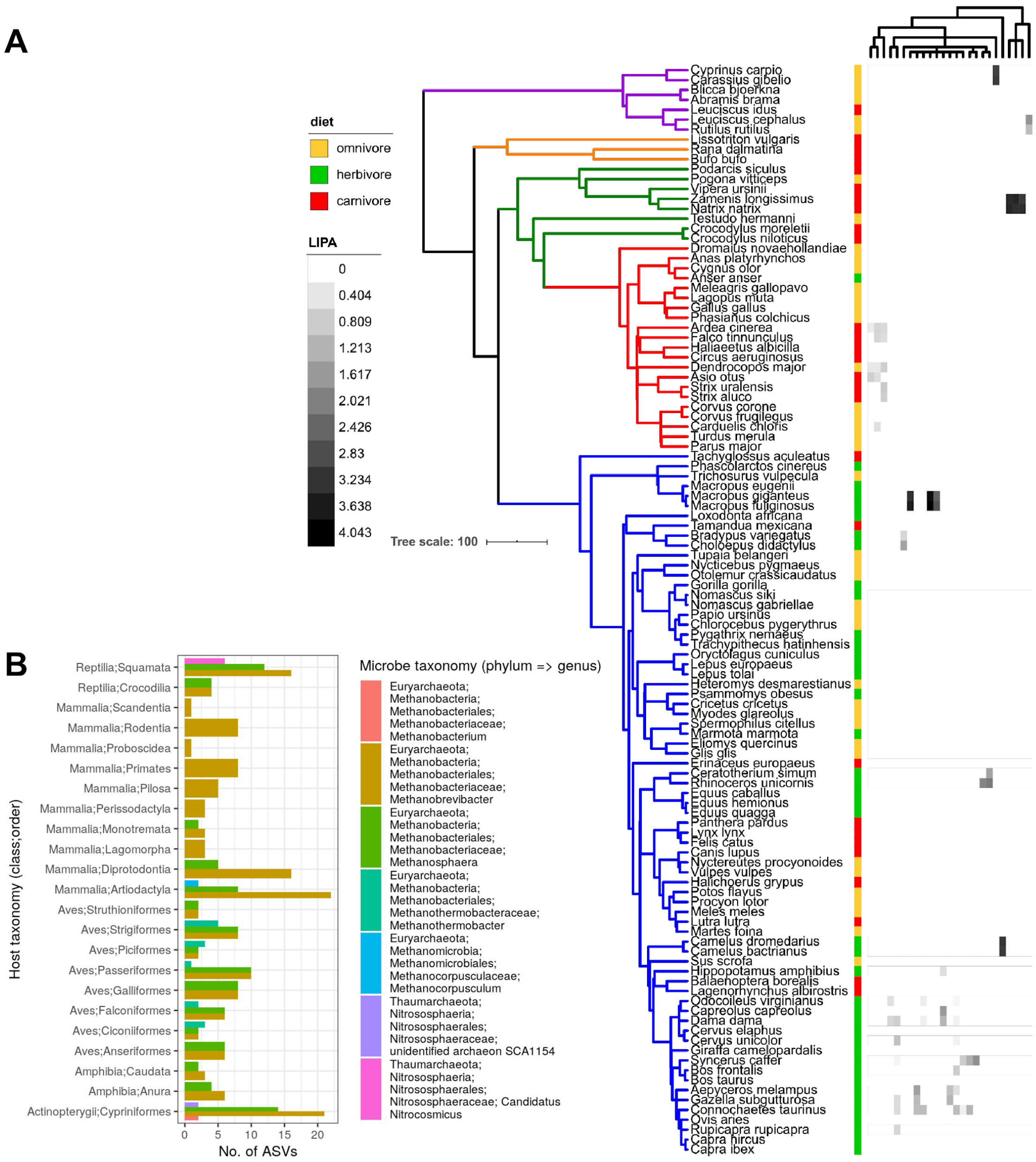

Figure S17. *The specific ASVs have similar abundances within certain vertebrate clades.* Various archaeal ASVs display local phylogenetic signal to various host clades. A) All ASVs with significant local phylogenetic signals (adj. $P < 0.05$) are mapped onto the host phylogeny. The phylogeny is the same as shown in Figure 1. The heatmap depicts local indicator of phylogenetic association (LIPA) values for each ASV–host association, with higher values indicating a stronger phylogenetic signal of ASV abundance. White boxes in the heatmap indicate non-significant LIPA tests. The dendrogram on the top of the heatmap is a cladogram based on taxonomy for each ASV (see Figure S18 for the full taxonomy). B) The bar plots show the number of ASVs with significant LIPA indices per archaeal genus and host clade. See the Statistical_source_data file for all other statistical information.

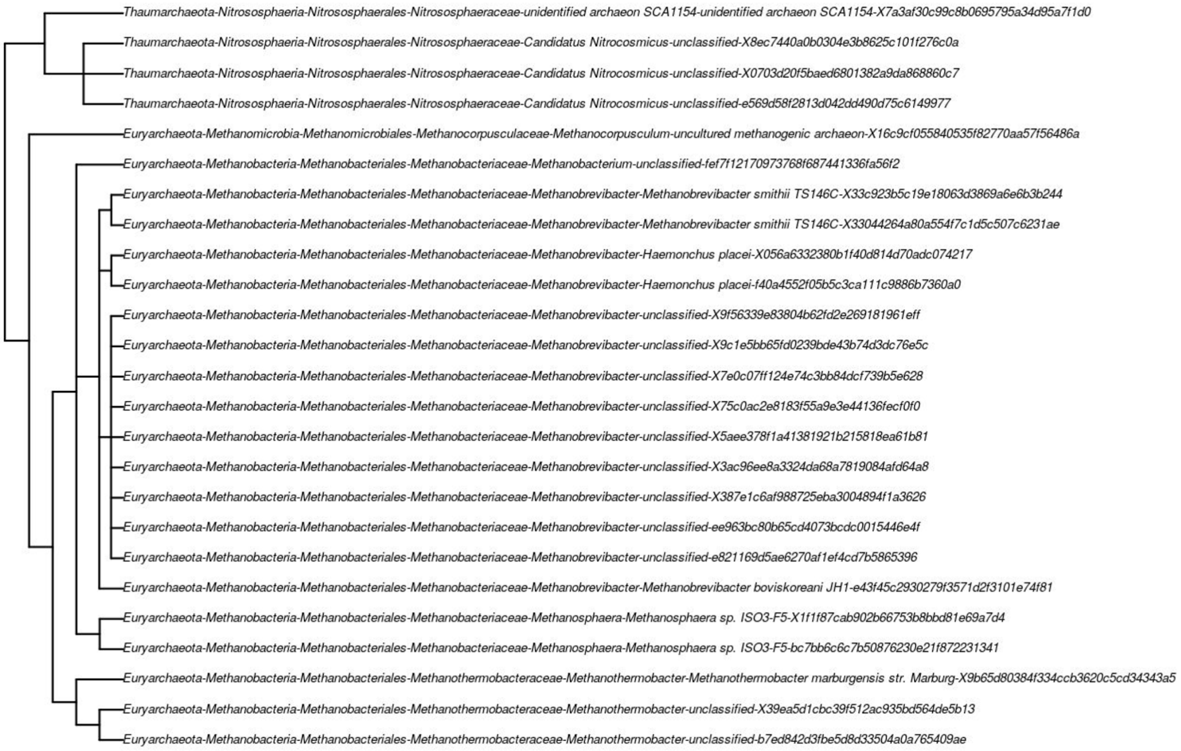

Figure S18. The cladogram as shown in Figure S17 with the entire ASV taxonomic classification as tip labels.

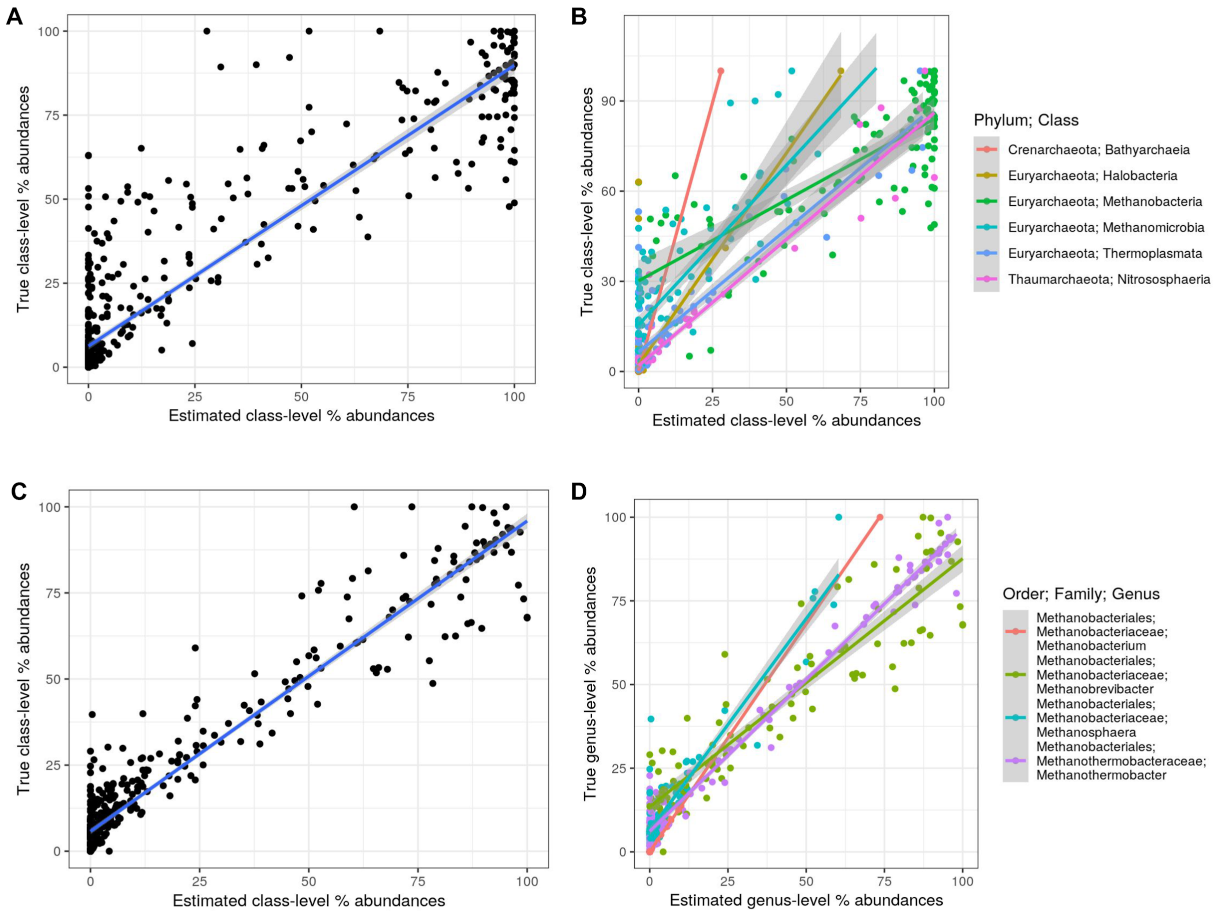

Figure S19. *Ancestral state reconstruction models accurately predict abundances in extant host species.* Linear regressions (colored lines) comparing ASR model predictions of archaeal abundances for each extant species relative to the observed mean abundance of all individuals per species. A) All class-level abundances, and B) abundances and linear regressions colored by class. C) All genus-level abundances for taxa belonging to *Methanobacteria*, and D) abundances and linear regressions colored by genus. Gray areas denote 95% confidence intervals for each linear model.

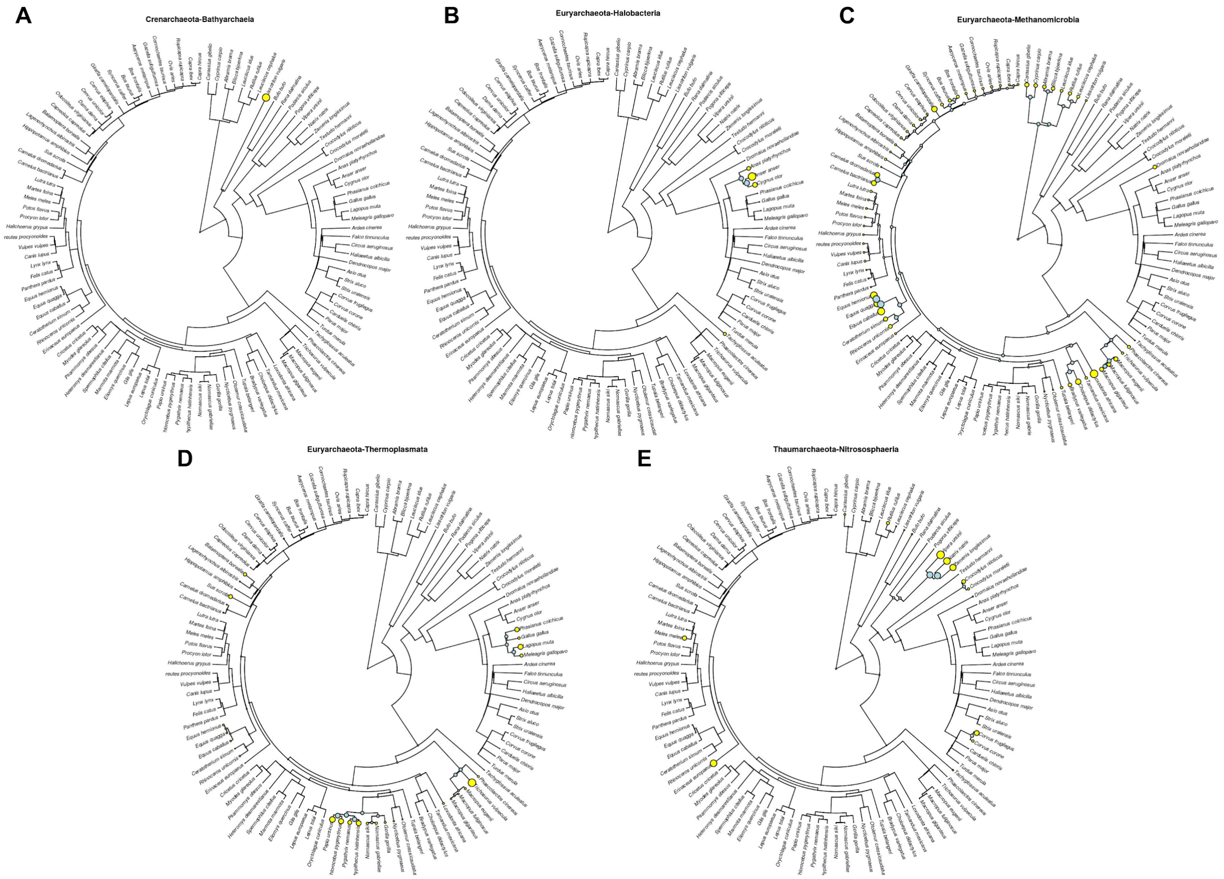

Figure S20. Predicted archaeal class-level abundance for extant host species (yellow circles) and and ancestral host species (blue circles): A) *Bathyarchaeia*, B) *Halobacteria*, C) *Methanomicrobia*, D) *Thermoplasmata*, and E) *Nitrososphaeria*. The phylogeny is the same as shown in Figure 1.

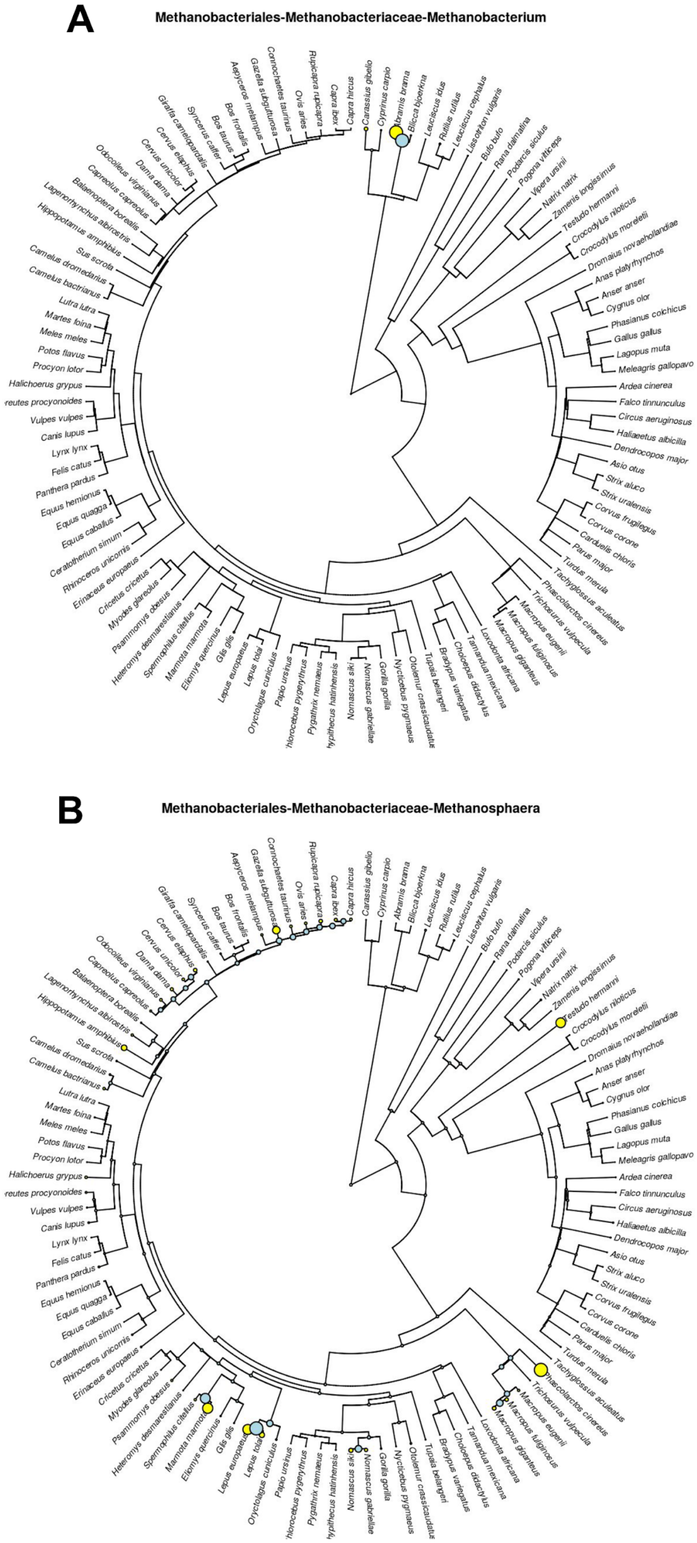

Figure S21. Predicted archaeal genus-level abundance for extant host species (yellow circles) and and ancestral host species (blue circles): A) *Methanobacterium* and B) *Methanosphaera*. The phylogeny is the same as shown in Figure 1.

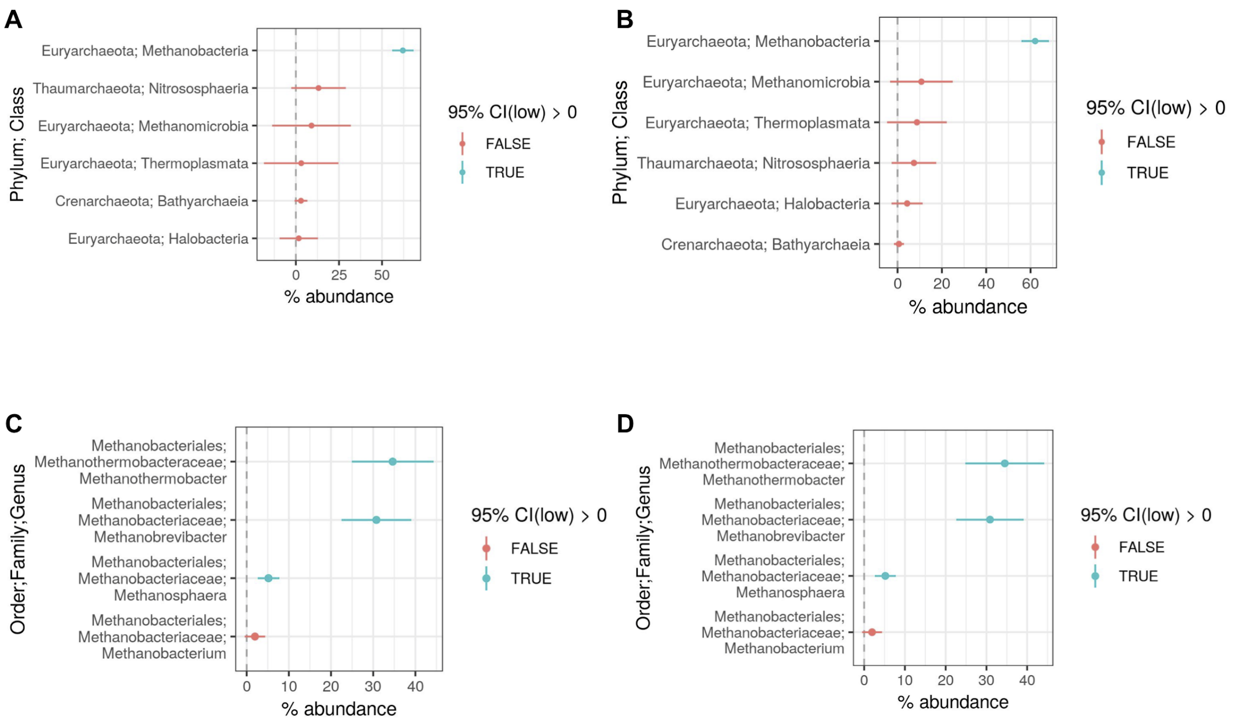

Figure S22. *The ASR estimations of archaeal abundances at the LCA of* Mammalia *and all 5 classes are robust to variable numbers of samples per host species.* The ASR relative abundances estimations are as shown in Figure 3, but the failed PCR/sequencing samples (Figure S2; Table S2) were included (all archaeal taxon abundances set to zero), and at most 3 samples were used per host species (147 samples; 110 host species). The points and line ranges denote the estimated taxon LCA abundance and 95% CIs, respectively. See the Statistical_source_data file for all other statistical information.

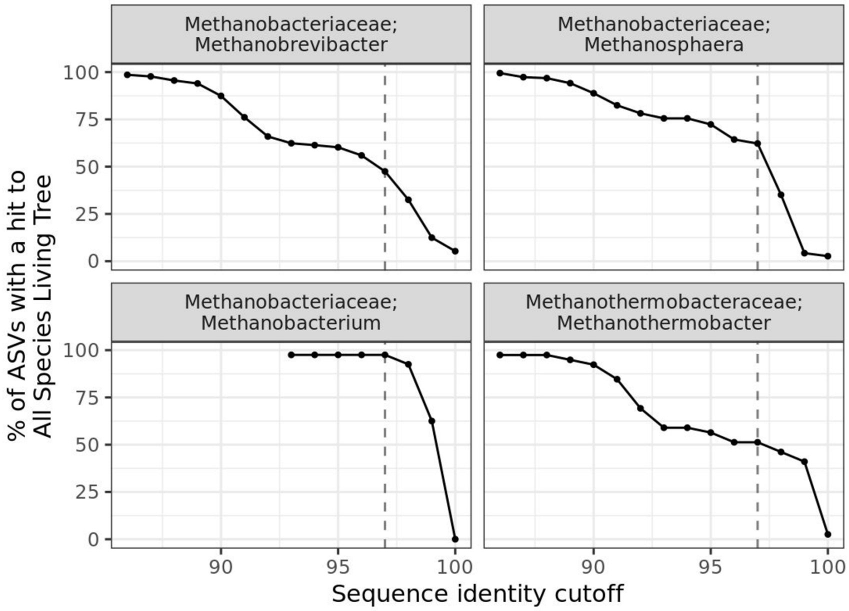

Figure S23. Methanobacteria *genera comprise a high proportion of uncultured ASVs.* Same as Figure S10, but just *Methanobacteria* genera. The plot facet labels are “family; genus”.

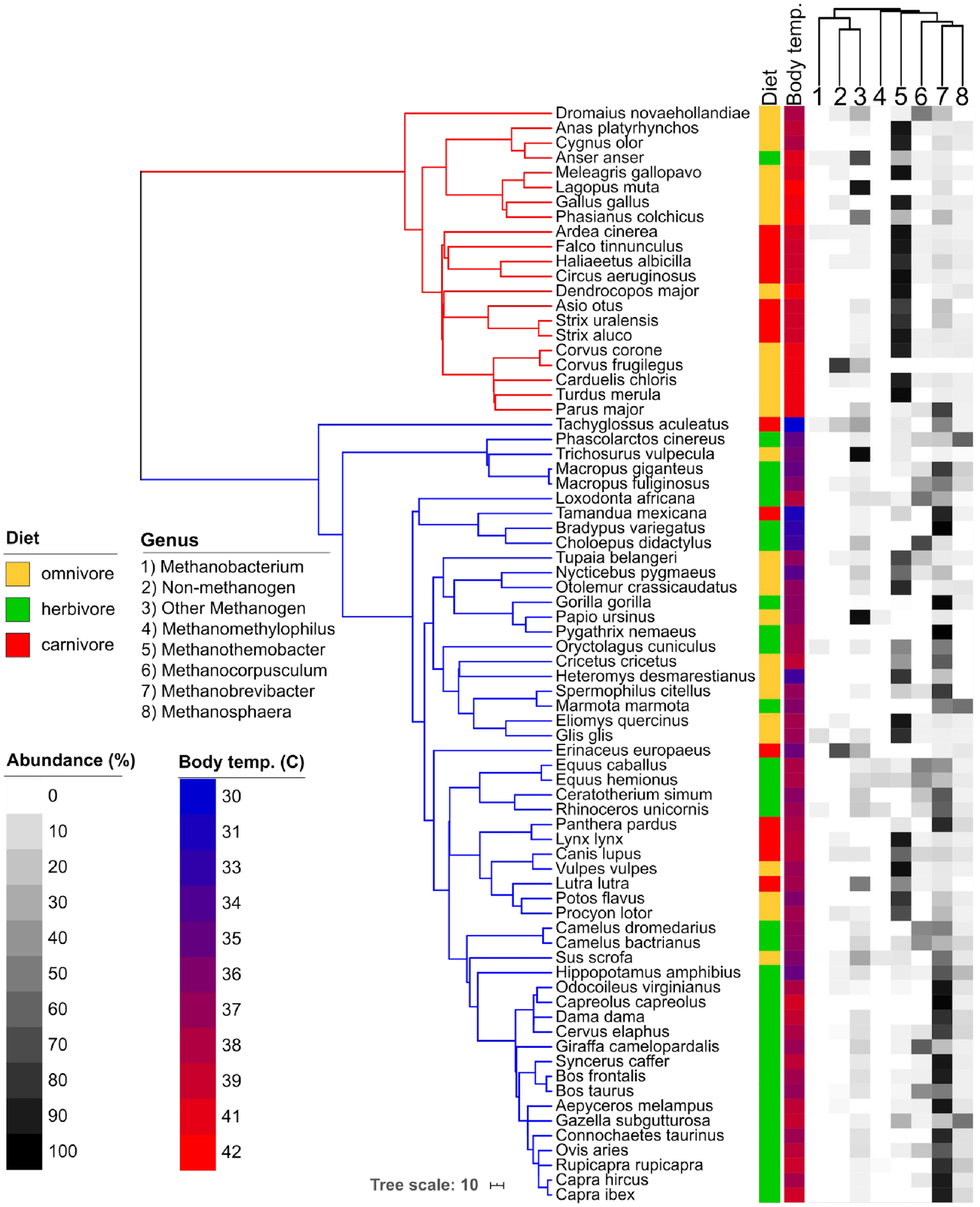

Figure S24. Methanothermobacter *is prevalent among avian species and associates with host body temperature.* The phylogeny is a pruned version (*n* = 74) of that shown in Figure 1. Host diet and body temperature are mapped into the tree along with genus-level archaeal abundances. The dendrogram above the heatmap is a cladogram depicting taxonomic relatedness. “Other Methanogen” refers to all other methanogen genera not specifically listed, and “Non-methanogen” refers to all non-methanogenic clades.

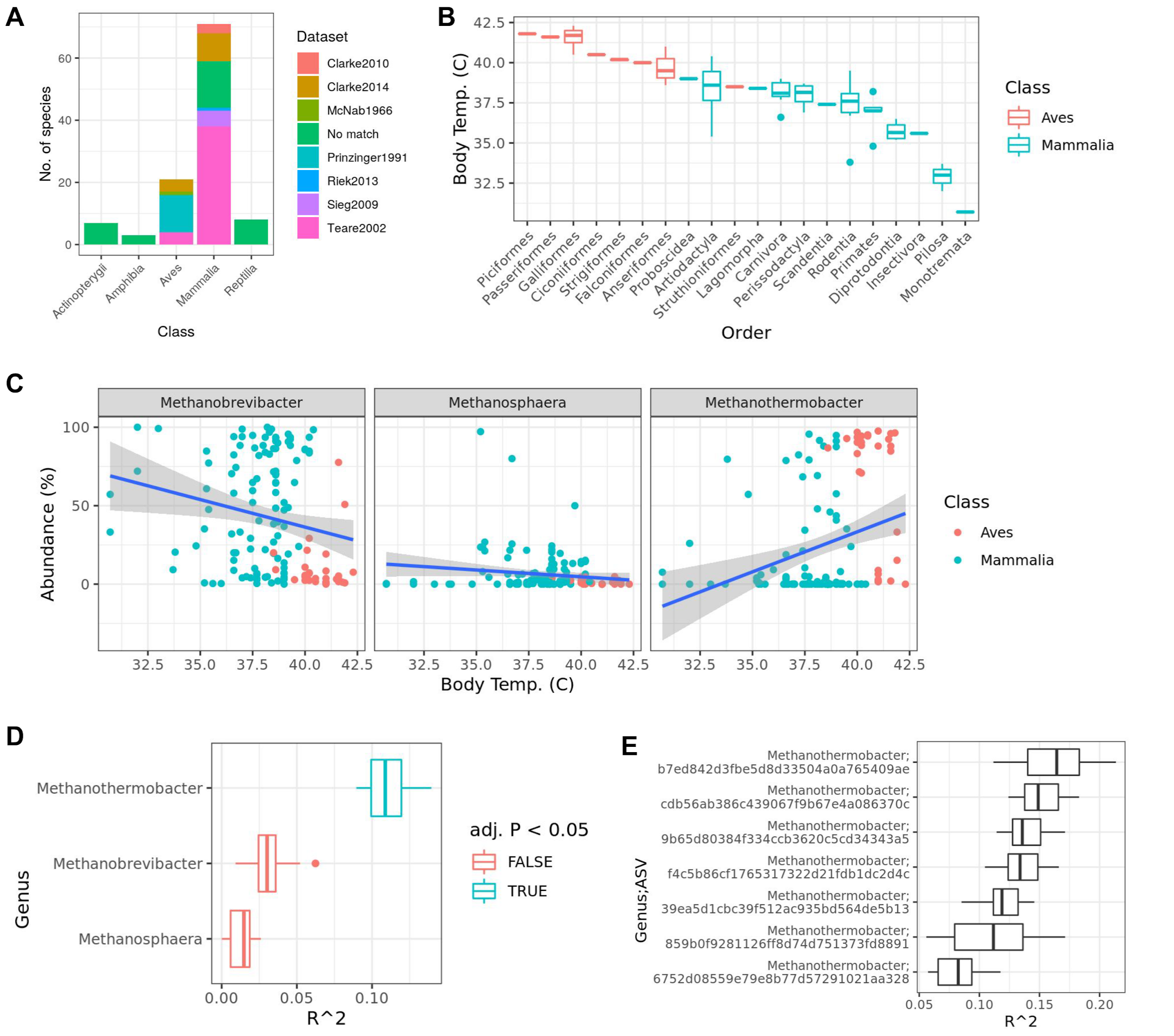

Figure S25. Methanothermobacter *abundance is explained by host body temperature*. A) The number of species with body temperature data, grouped by the body temperature dataset (see also Table S7). B) The distribution of body temperatures per host taxonomic order (one data point per species). C) Relative abundances of *Methanobacteria* genera as a function of host body temperature (celcius). The blue lines denote linear regressions with 95% CIs represented by the grey zones. D) RRPP coefficients of genus-level abundances as a linear function of host body temperature. Boxplots show the distribution across 100 permutations (*P*-values: *Methanothermobacter* = 0.2, *Methanobrevibacter* = 0.02, *Methanosphaera* = 0.01).
E) The same as D, but ASV-level abundances used, with only significant ASVs shown. Note that host phylogeny was not used for the RRPP models shown in D & E. No taxa were significant when accounting for host phylogeny. Box centerlines, edges, whiskers, and points signify the median, interquartile range (IQR), 1.5 × IQR, and >1.5 × IQR, respectively. See the Statistical_source_data file for all other statistical information.

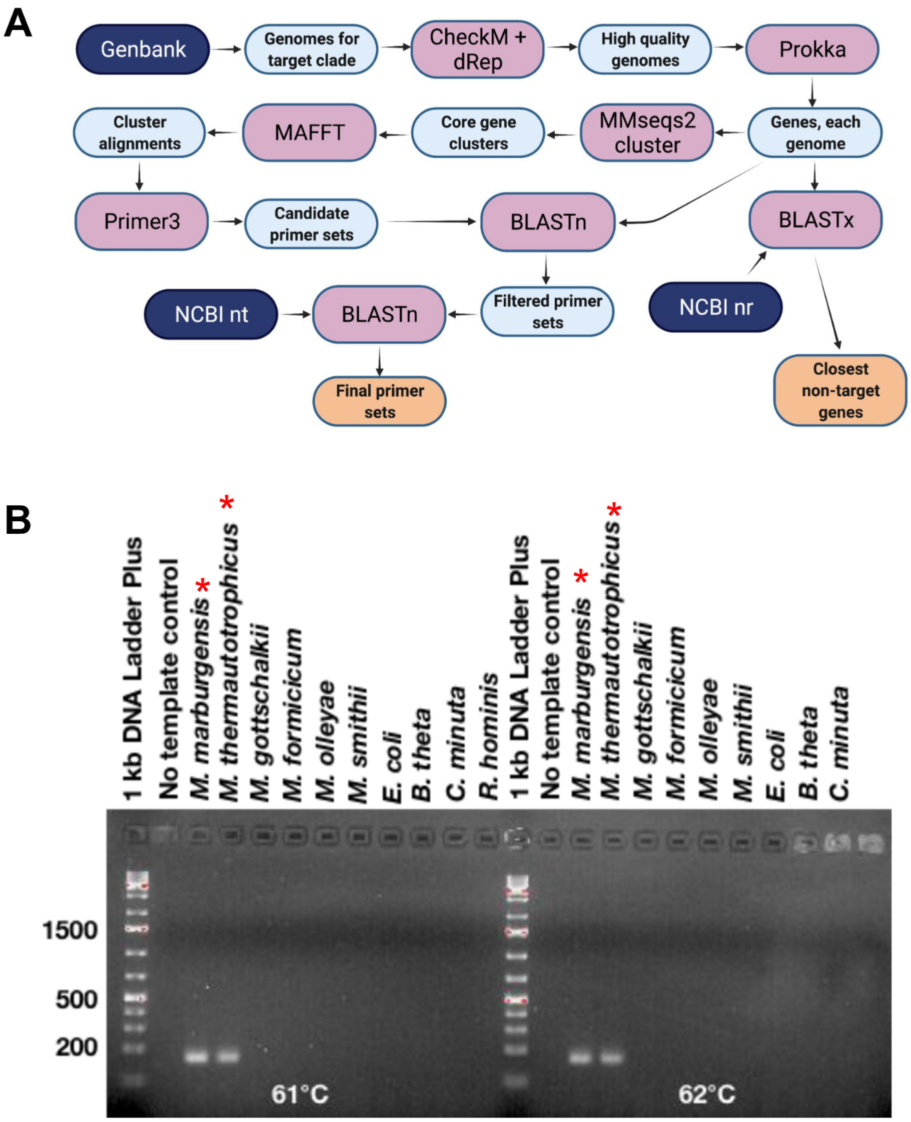

Figure S26. *qPCR primers designed with Core Genome Primers (CGP) are specific to* Methanothermobacter. A) The general workflow of CGP. Dark blue, pink, light blue, and orange ovals denote public databases, software, intermediate data, and final data, respectively. The diagram was created with BioRender.com. B) A 2% agarose gel showing PCR products for a screen of the *Methanothermobacter*-targeting 174FR primers against a gDNA panel of various target and nontarget isolates. The red asterisks mark the 2 *Methanothermobacter* isolates included in the gDNA panel. The temperatures listed at the bottom of the gel denote the PCR melting temperatures used. The full name of each isolate, as ordered in the gel image, is as follows: *Methanothermobacter marburgensis DSM-2133*, *Methanothermobacter thermautotrophicus DSM-1053*, *Methanobrevibacter gottschalkii DSM-11977*, *Methanobacterium formicicum DSM-1535*, *Methanobrevibacter olleyae DSM-16632*, *Methanobrevibacter smithii DSM-861*, *Escherichia coli NEB 5-alpha*, *Bacteroides thetaiotaomicron VPI-5482*, *Christensenella minuta DSM-22607*, and *Roseburia hominis DSM-16839*.

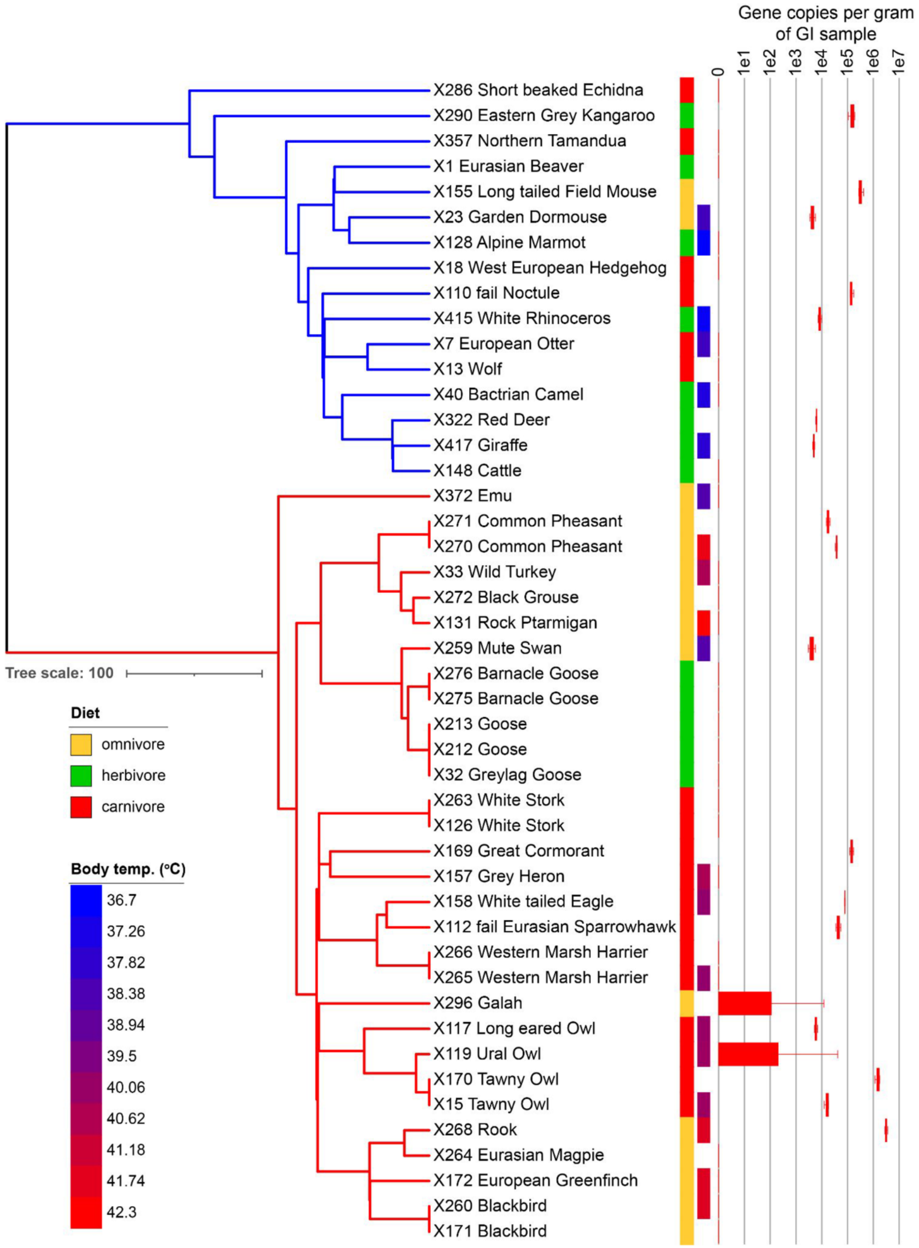

Figure S27. *qPCR with the* Methanothermobacter-*targeting primers "174FR" suggests clade presence in many avian and mammalian species*. The phylogeny is a pruned version of the tree shown in Figure S8. The 2 color strips adjacent to the tree are host diet and body temperature, respectively (as shown in Figure S24). The box plots denote the value distribution across 3 PCR replicates. The primers "174FR" were designed to target a single copy core gene of the *Methanothermobacter* pan-genome (see Figure S26 and Methods). Box centerlines, edges, whiskers, and points signify the median, interquartile range (IQR), 1.5 × IQR, and >1.5 × IQR, respectively. The total sample size is 46.

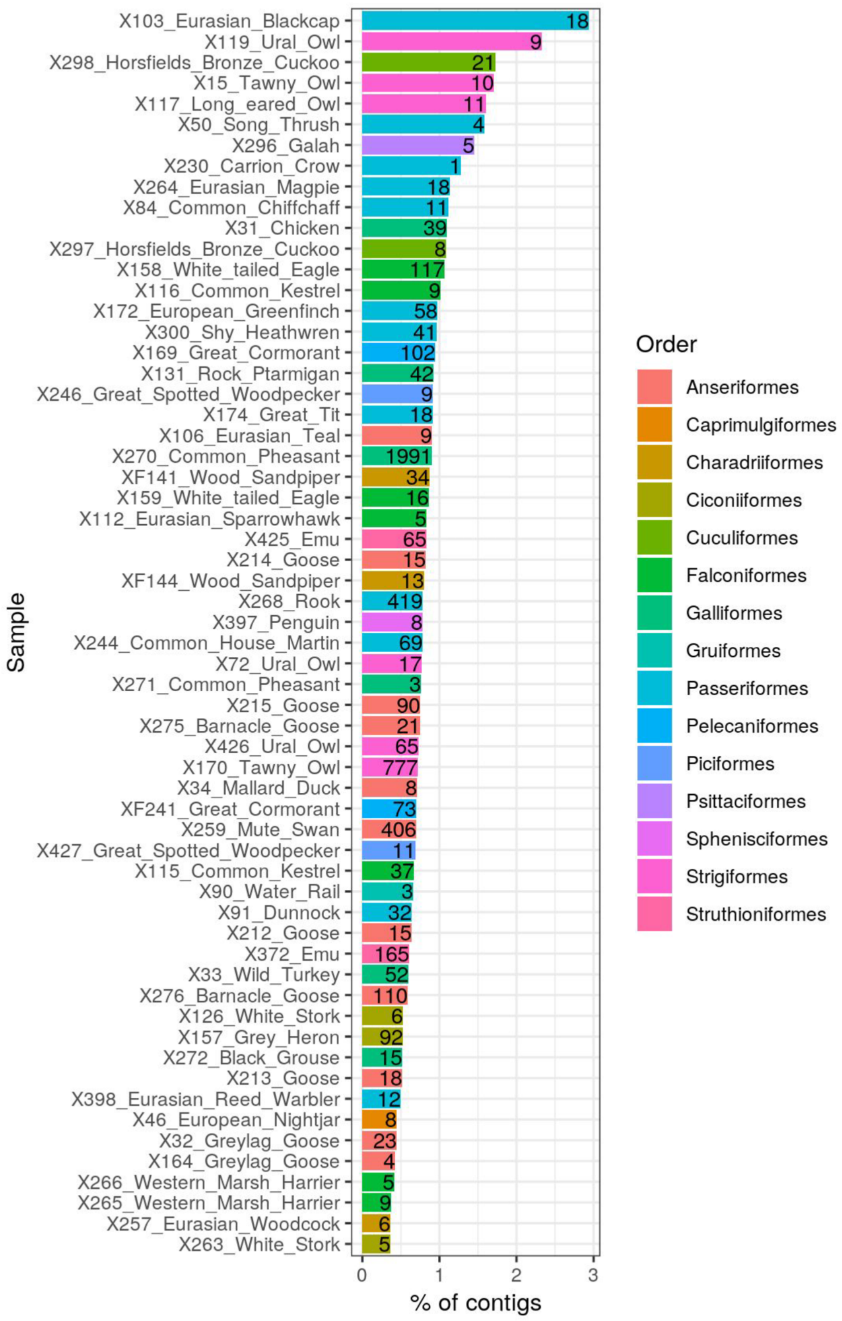

Figure S28. Methanothermobacter *contigs detected in avian metagenomes.* Contigs of ≥1.5 kb were mapped to *Methanothermobaceteriales* species representative genomes (*n* = 94) via Minimap2. Contigs mapping to multiple species or with a query coverage (alignment length / query length) of < 0.9 were filtered. The bars show the percent of total assembled contigs that map to *Methanothermobacteraceae* or *Methanothermobacteraceae A* species (GTDB Release 95 taxonomy). The numbers on each bar denote the number contigs that mapped to the target taxa.

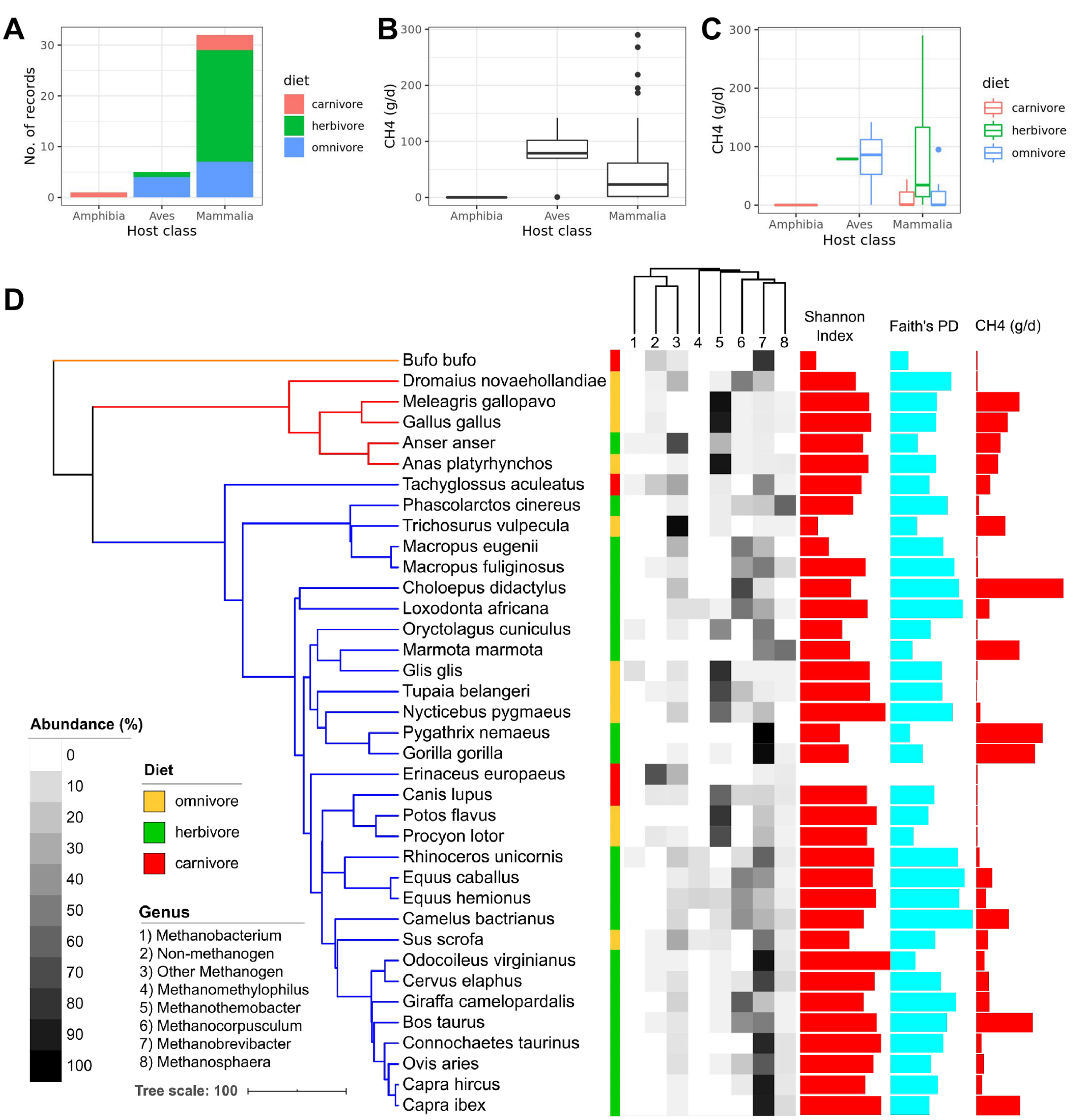

Figure S29. *Published animal methane emission data indicates that the avian species dominated by* Methanothermobacter *emit substantial amounts of methane.* A) The number of records obtained from Hackstein & van Alen 1996 (*n* = 27) and Clauss et al., 2020 (*n* = 10), grouped by host class and diet. B) & C) the distribution of methane emission rates per host species, grouped by class and C) colored by host diet. D) The phylogeny is a pruned version of that shown in Figure 1. From left to right, the data mapped onto the phylogeny is: host diet, methanogen genus mean abundances, methanogen ASV diversity (Shannon Index & Faith's PD), and methane emission rates. The lack of diversity values for *Erinaceus europaeus* (European hedgehog) is due to an absence of detectable methanogen ASVs. Box centerlines, edges, whiskers, and points signify the median, interquartile range (IQR), 1.5 × IQR, and >1.5 × IQR, respectively.

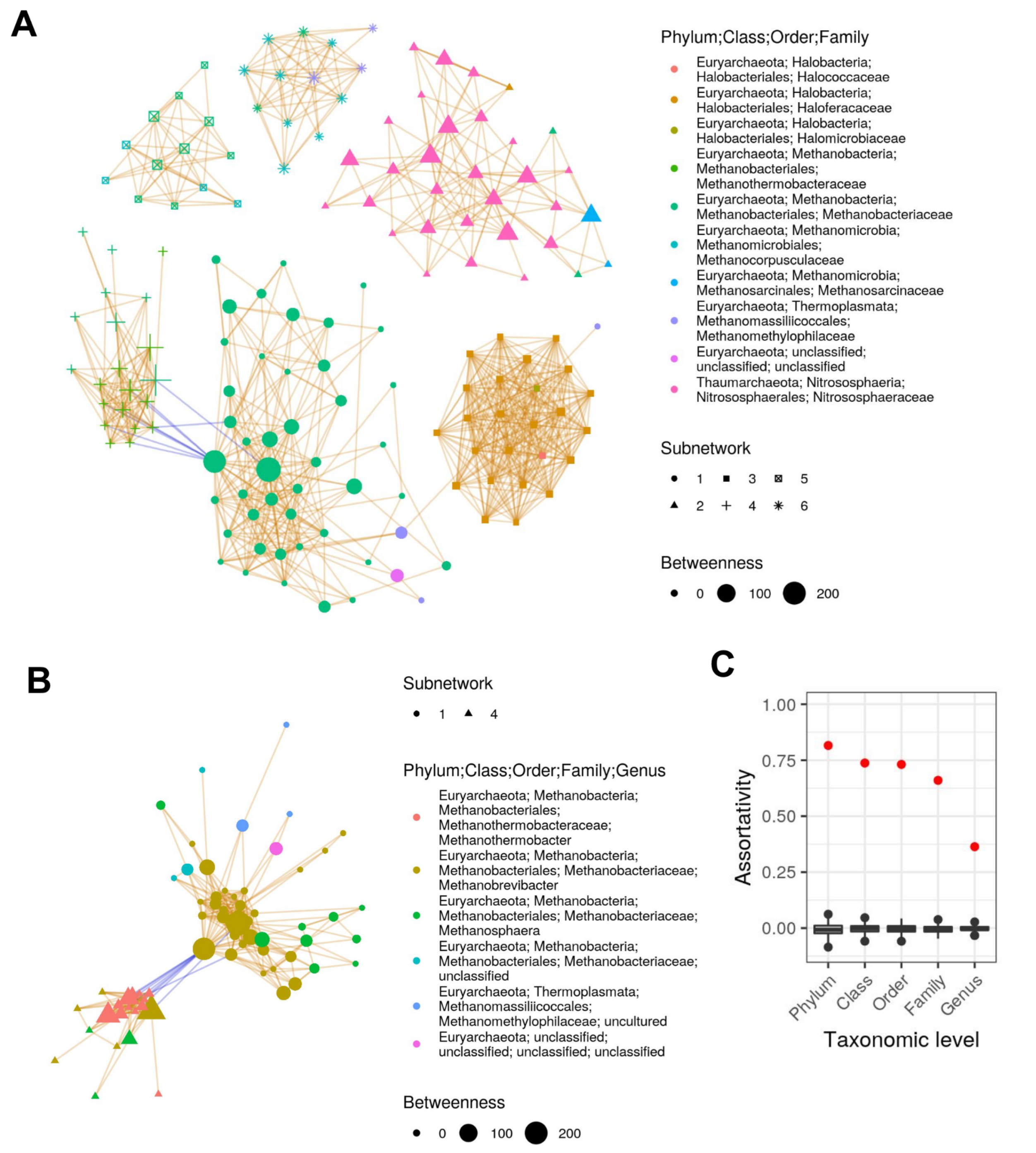

Figure S30. *Archaeal ASVs generally co-occur with members of the same taxonomic group.* The network nodes represent ASVs, with color denoting family-level taxonomic classifications, and shape denoting subnetwork (defined by clustering the network with the walktrap algorithm). Edges represent significant positive and negative co-occurrences among ASVs as denoted by orange and blue edges, respectively. Node size represents “betweenness”, which is a measure of node connectedness. For clarity, only the largest 6 subnetworks are shown (but see Figure

S31). B) Only subnetworks 1 and 4 are shown with node colors denoting genus-level classifications. C) The assortativity of ASVs by taxonomic level, in which a value of 1 means that all connected ASVs belong to the same taxonomic group, while a value of 0 denotes random association, and negative values indicate a dominance of inter-clade associations. The red points are the observed values, while the boxplots denote values for 100 permutations of networks with the same number of nodes and edges as the true network, but edges were randomly assigned. Box centerlines, edges, whiskers, and points signify the median, interquartile range (IQR), 1.5 × IQR, and >1.5 × IQR, respectively.

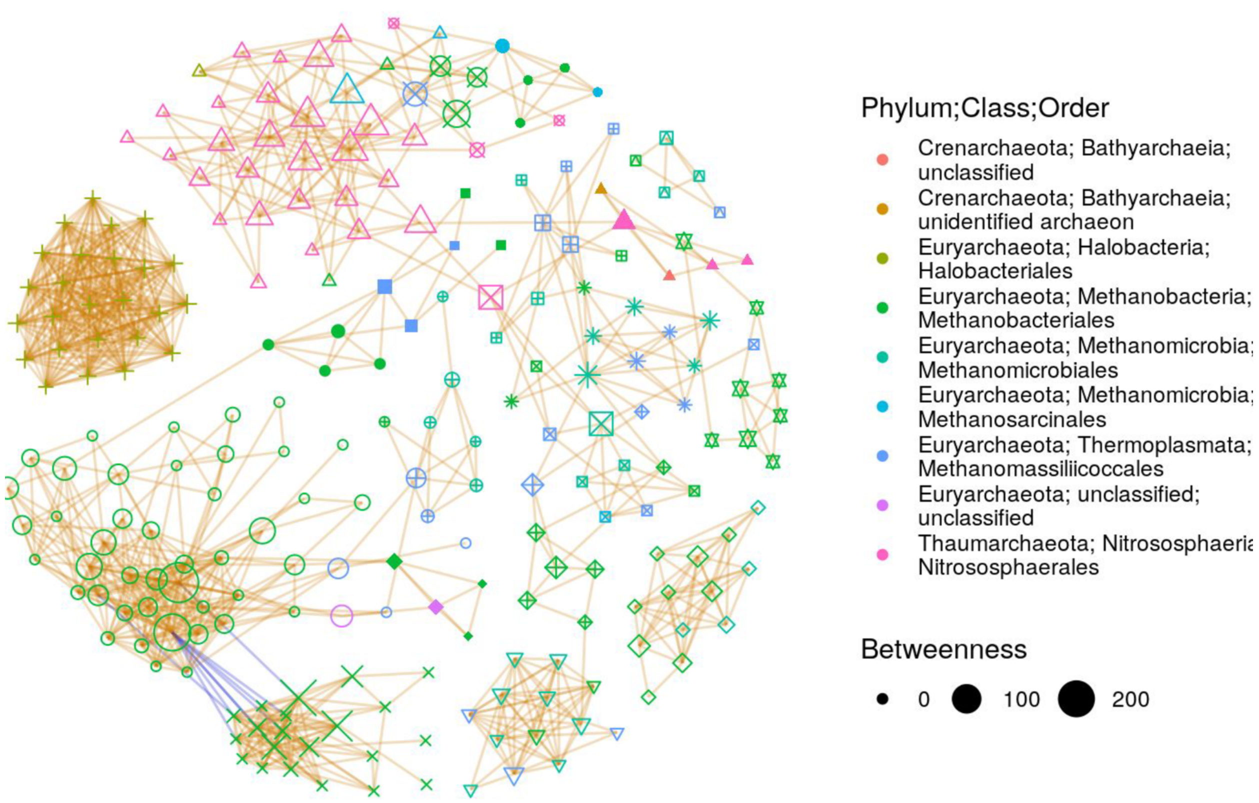

Figure S31. The same co-occurrence network as shown in Figure S30, but the largest 19 subnetworks are shown (238 of 313 ASVs) instead of just the largest 6 (151 of 313 ASVs). The entire co-occurrence network comprised 96 subnetworks, but to be able to distinguish among shapes denoting network nodes, only the top 19 subnetworks are shown.

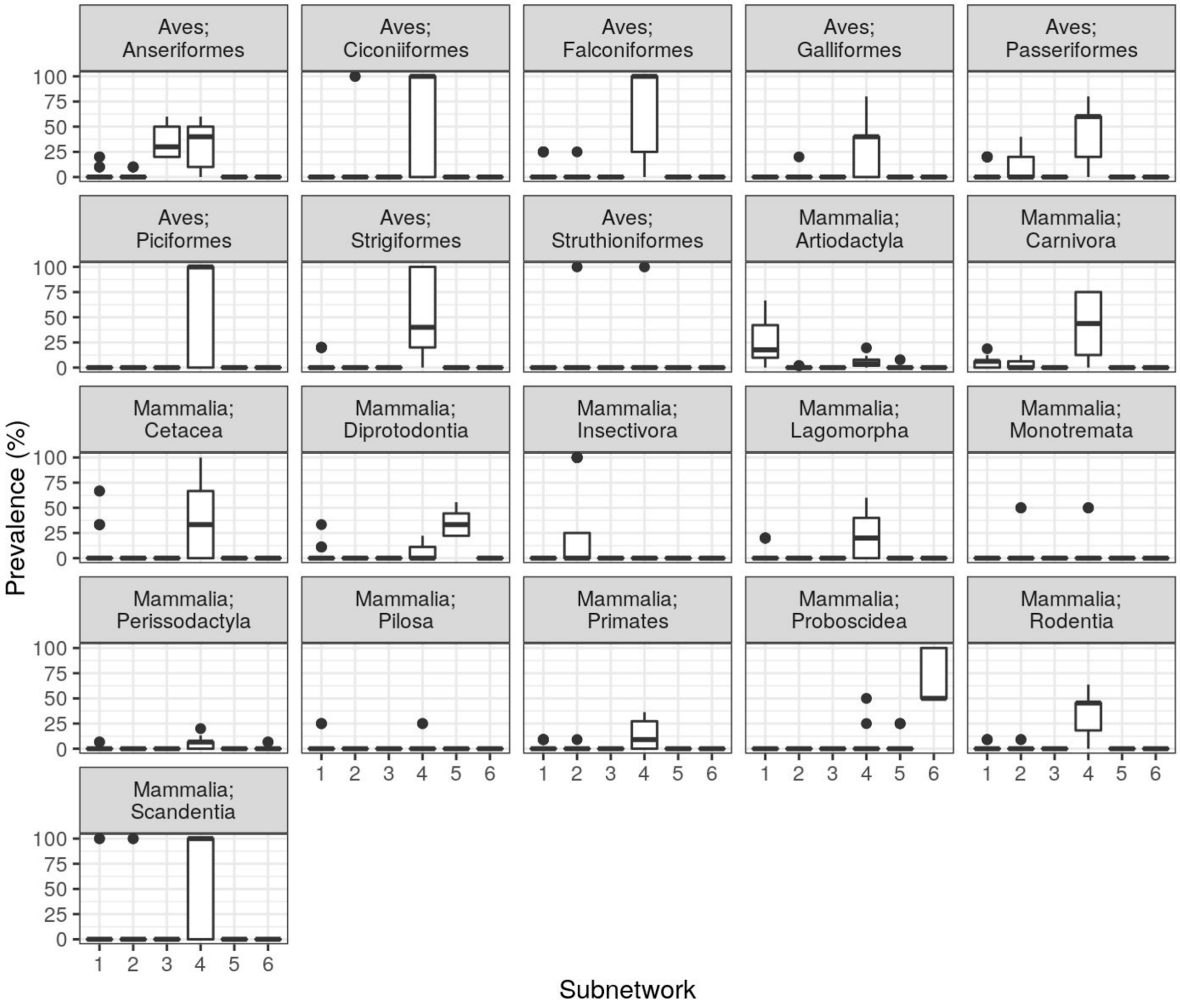

Figure S32. The percent of samples in which each ASV was observed (prevalence), grouped by the subnetwork to which each ASV belongs (see Figure S30A) and faceted by host taxonomic order. Box centerlines, edges, whiskers, and points signify the median, interquartile range (IQR), 1.5 × IQR, and >1.5 × IQR, respectively. The total sample size (i.e., number of hots) is 165.

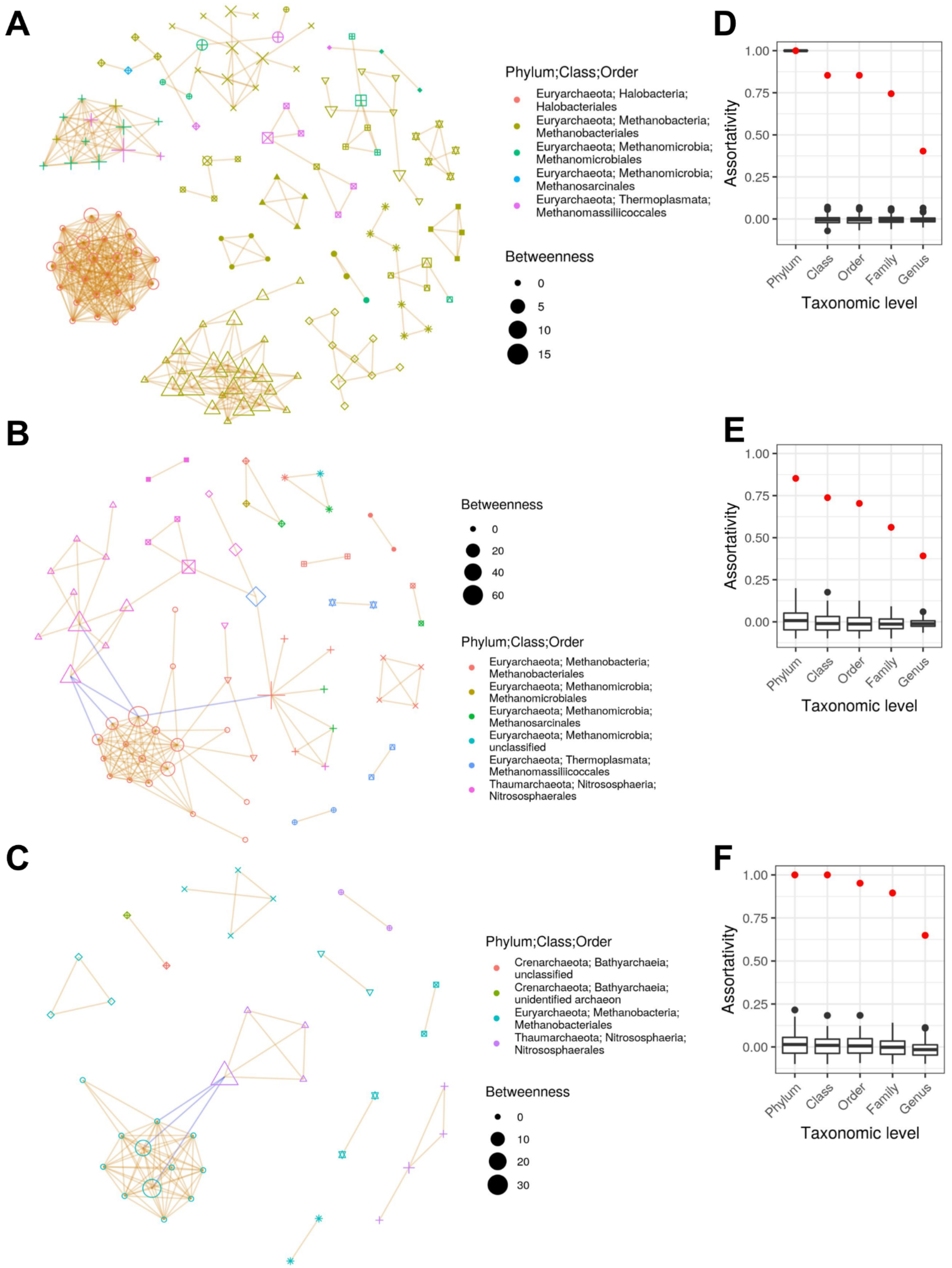
A
Phylum;Class;Order
Euryarchaeota; Halobacteria; Halobacteriales
Euryarchaeota; Methanobacteria; Methanobacteriales
Euryarchaeota; Methanomicrobia; Methanomicrobiales
Euryarchaeota; Methanomicrobia; Methanosarcinales
Euryarchaeota; Thermoplasmata; Methanomassiliicoccales
Betweenness
0
5
10
15
D
Assortativity
1.00
0.75
0.50
0.25
0.00
Phylum
Class
Order
Family
Genus
Taxonomic level
B
Betweenness
0
20
40
60
Phylum;Class;Order
Euryarchaeota; Methanobacteria; Methanobacteriales
Euryarchaeota; Methanomicrobia; Methanomicrobiales
Euryarchaeota; Methanomicrobia; Methanosarcinales
Euryarchaeota; Methanomicrobia; unclassified
Euryarchaeota; Thermoplasmata; Methanomassiliicoccales
Thaumarchaeota; Nitrososphaeria; Nitrososphaerales
E
Assortativity
1.00
0.75
0.50
0.25
0.00
Phylum
Class
Order
Family
Genus
Taxonomic level
C
Phylum;Class;Order
Crenarchaeota; Bathyarchaeia; unclassified
Crenarchaeota; Bathyarchaeia; unidentified archaeon
Euryarchaeota; Methanobacteria; Methanobacteriales
Thaumarchaeota; Nitrososphaeria; Nitrososphaerales
Betweenness
0
10
20
30
F
Assortativity
1.00
0.75
0.50
0.25
0.00
Phylum
Class
Order
Family
Genus
Taxonomic level

Figure S33. Co-occurrence networks for A) just herbivore, B) just omnivore, C) just carnivore samples. Node size represents "betweenness", which is a measure of node connectedness. Orange and blue edges denote significant positive and negative co-occurrences, respectively. D-F) Assortativity of nodes the graph, determined for each taxonomic level from phylum to genus. High assortativity values indicate that the co-occurring taxa largely belong to the same taxonomic group. The boxplots show the distribution of assortativity values obtained from 100 random permutations of the network (same number of nodes and edges, but edges randomly assigned). The red points denote the assortativity values for the true networks shown in plots A-C. Box centerlines, edges, whiskers, and points signify the median, interquartile range (IQR), 1.5 × IQR, and >1.5 × IQR, respectively. The sample sizes for plots D-F are 100 (i.e., 100 random permutations).

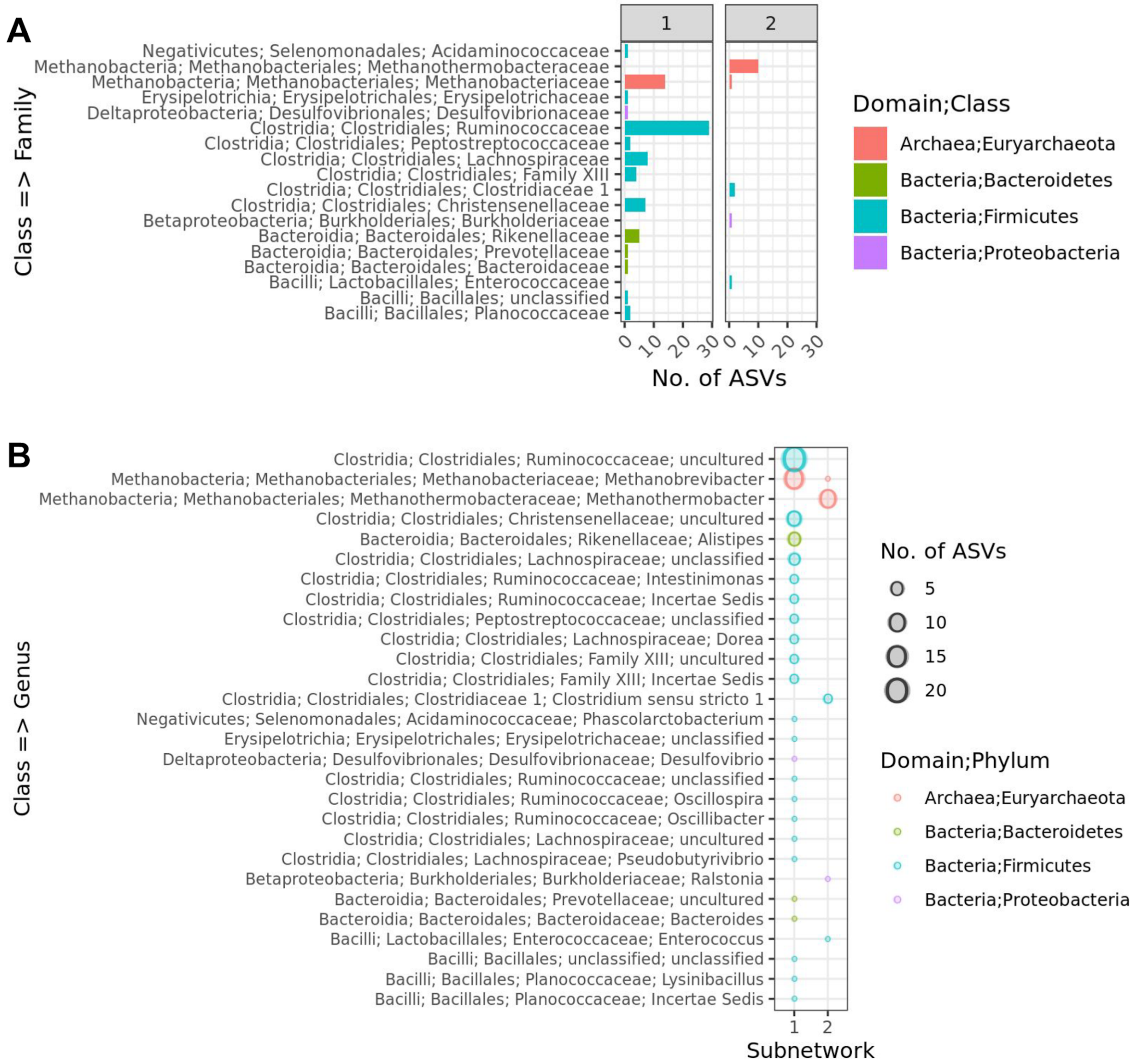

Figure S34. Taxonomic composition of the 2 sub-networks (Figure 4D) containing archaeal ASVs, with the number of ASVs summarized at the A) family and B) genus taxonomic levels.

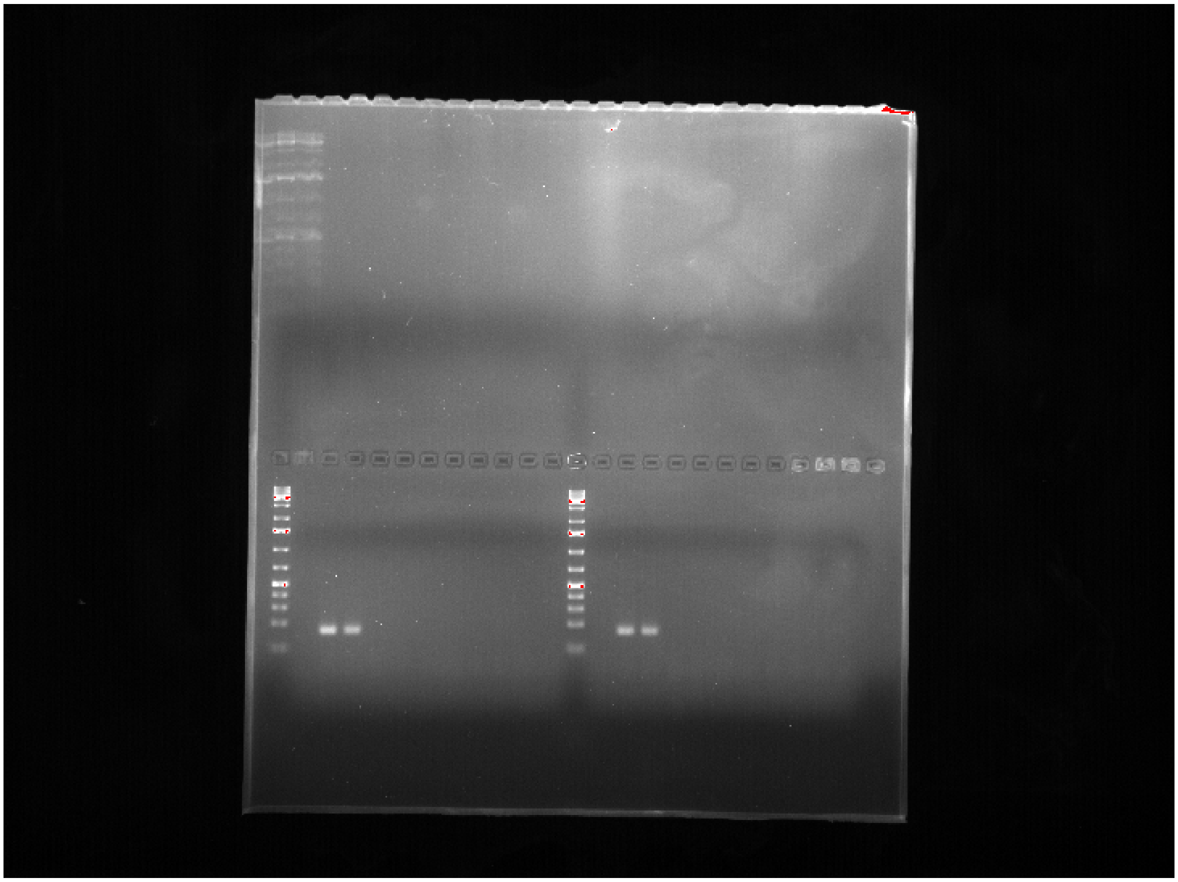

Figure S35. The unedited gel image corresponding to the image shown in Figure S26B.

Supplemental References

Bonin, Adam S., and David R. Boone. “The Order Methanobacteriales.” In *The Prokaryotes*, edited by Martin Dworkin Professor Dr., Stanley Falkow, Eugene Rosenberg, Karl-Heinz Schleifer, and Erko Stackebrandt, 231–43. Springer New York, 2006.

Borrel, Guillaume, Jean-François Brugère, Simonetta Gribaldo, Ruth A. Schmitz, and Christine Moissl-Eichinger. “The Host-Associated Archaeome.” *Nature Reviews. Microbiology*, July 20, 2020, 1–15.

Clarke, Andrew, and Mary I. O’Connor. “Diet and Body Temperature in Mammals and Birds.” *Global Ecology and Biogeography: A Journal of Macroecology* 23, no. 9 (September 26, 2014): 1000–1008.

Clarke, Andrew, Peter Rothery, and Nick J. B. Isaac. “Scaling of Basal Metabolic Rate with Body Mass and Temperature in Mammals.” *The Journal of Animal Ecology* 79, no. 3 (May 2010): 610–19.

Clauss, M., M. T. Dittmann, C. Vendl, K. B. Hagen, S. Frei, S. Ortmann, D. W. H. Müller, et al. “Review: Comparative Methane Production in Mammalian Herbivores.” *Animal: An International Journal of Animal Bioscience* 14, no. S1 (March 2020): s113–23.

Collyer, Michael L., and Dean C. Adams. “RRPP: An R Package for Fitting Linear Models to High-dimensional Data Using Residual Randomization.” Edited by Robert Freckleton. *Methods in Ecology and Evolution / British Ecological Society* 9, no. 7 (July 29, 2018): 1772–79.

Goodrich, Julia K., Jillian L. Waters, Angela C. Poole, Jessica L. Sutter, Omry Koren, Ran Blekhman, Michelle Beaumont, et al. “Human Genetics Shape the Gut Microbiome.” *Cell* 159, no. 4 (November 6, 2014): 789–99.

Hackstein, Johannes H. P., and Theo A. van Alen. “Fecal Methanogens and Vertebrate Evolution.” *Evolution; International Journal of Organic Evolution* 50, no. 2 (1996): 559–72.

Hansen, Elizabeth E., Catherine A. Lozupone, Federico E. Rey, Meng Wu, Janaki L. Guruge, Aneesha Narra, Jonathan Goodfellow, et al. “Pan-Genome of the Dominant Human Gut-Associated Archaeon, Methanobrevibacter Smithii, Studied in Twins.” *Proceedings of the National Academy of Sciences of the United States of America* 108 Suppl 1, no. Supplement 1 (March 15, 2011): 4599–4606.

Hutchinson, Matthew C., E. Fernando Cagua, Juan A. Balbuena, Daniel B. Stouffer, and Timothée Poisot. “Paco: Implementing Procrustean Approach to Cophylogeny in R.” Edited by Richard Fitzjohn. *Methods in Ecology and Evolution / British Ecological Society* 8, no. 8 (August 27, 2017): 932–40.

Koskinen, Kaisa, Manuela R. Pausan, Alexandra K. Perras, Michael Beck, Corinna Bang, Maximilian Mora, Anke Schilhabel, Ruth Schmitz, and Christine Moissl-Eichinger. “First Insights into the Diverse Human Archaeome: Specific Detection of Archaea in the Gastrointestinal Tract, Lung, and Nose and on Skin.” *mBio* 8, no. 6 (November 14, 2017). doi:10.1128/mBio.00824-17.

McNab, Brian K. “The Metabolism of Fossorial Rodents: A Study of Convergence.” *Ecology* 47, no. 5 (September 1, 1966): 712–33.

Paradis, Emmanuel, Julien Claude, and Korbinian Strimmer. “APE: Analyses of Phylogenetics and Evolution in R Language.” *Bioinformatics* 20, no. 2 (January 22, 2004): 289–90.

Pausan, Manuela R., Cintia Csorba, Georg Singer, Holger Till, Veronika Schöpf, Elisabeth Santigli, Barbara Klug, Christoph Högenauer, Marcus Blohs, and Christine Moissl-Eichinger. “Exploring the Archaeome: Detection of Archaeal Signatures in the Human Body.” *Frontiers in Microbiology* 10 (December 5, 2019): 2796.

Prinzinger, R., A. Preßmar, and E. Schleucher. “Body Temperature in Birds.” *Comparative Biochemistry and Physiology. Part A, Physiology* 99, no. 4 (January 1, 1991): 499–506.

Raymann, Kasie, Andrew H. Moeller, Andrew L. Goodman, and Howard Ochman. “Unexplored

Archaeal Diversity in the Great Ape Gut Microbiome." *mSphere* 2, no. 1 (January 2017). doi:10.1128/mSphere.00026-17.
Revell, Liam J. "Phylogenetic Signal and Linear Regression on Species Data: Phylogenetic Regression." *Methods in Ecology and Evolution / British Ecological Society* 1, no. 4 (December 30, 2010): 319–29.
Riek, Alexander, and Fritz Geiser. "Allometry of Thermal Variables in Mammals: Consequences of Body Size and Phylogeny." *Biological Reviews of the Cambridge Philosophical Society* 88, no. 3 (August 2013): 564–72.
Sieg, Annette E., Michael P. O'Connor, James N. McNair, Bruce W. Grant, Salvatore J. Agosta, and Arthur E. Dunham. "Mammalian Metabolic Allometry: Do Intraspecific Variation, Phylogeny, and Regression Models Matter?" *The American Naturalist* 174, no. 5 (November 2009): 720–33.
Söllinger, Andrea, and Tim Urich. "Methylotrophic Methanogens Everywhere - Physiology and Ecology of Novel Players in Global Methane Cycling." *Biochemical Society Transactions*, December 10, 2019. doi:10.1042/BST20180565.
Teare, Andrew. "International Species Information System, Medical Animal Records Keeping System (MedARKS) 2002 Data Extraction," 2002. https://www.species360.org/about-us/mission-history/.
Thompson, Luke R., Jon G. Sanders, Daniel McDonald, Amnon Amir, Joshua Ladau, Kenneth J. Locey, Robert J. Prill, et al. "A Communal Catalogue Reveals Earth's Multiscale Microbial Diversity." *Nature* 551, no. 7681 (November 23, 2017): 457–63.
Walters, William, Embriette R. Hyde, Donna Berg-Lyons, Gail Ackermann, Greg Humphrey, Alma Parada, Jack A. Gilbert, et al. "Improved Bacterial 16S rRNA Gene (V4 and V4-5) and Fungal Internal Transcribed Spacer Marker Gene Primers for Microbial Community Surveys." *mSystems* 1, no. 1 (January 2016). doi:10.1128/mSystems.00009-15.
Youngblut, Nicholas D., Jacobo de la Cuesta-Zuluaga, Georg H. Reischer, Silke Dauser, Nathalie Schuster, Chris Walzer, Gabrielle Stalder, Andreas H. Farnleitner, and Ruth E. Ley. "Large-Scale Metagenome Assembly Reveals Novel Animal-Associated Microbial Genomes, Biosynthetic Gene Clusters, and Other Genetic Diversity." *mSystems* 5, no. 6 (December 22, 2020). doi:10.1128/mSystems.01045-20.
Youngblut, Nicholas D., Georg H. Reischer, William Walters, Nathalie Schuster, Chris Walzer, Gabrielle Stalder, Ruth E. Ley, and Andreas H. Farnleitner. "Host Diet and Evolutionary History Explain Different Aspects of Gut Microbiome Diversity among Vertebrate Clades." *Nature Communications* 10, no. 1 (May 16, 2019): 2200.
