# Supplementary material for: Vertebrate host phylogeny influences gut archaeal diversity
Source: Nat Microbiol. 2021 Oct 26;6(11):1443–54. doi: 10.1038/s41564-021-00980-2 (PMC8556154; doi:10.1038/s41564-021-00980-2)
Supplement: Supplementary file 2 — Reporting Summary [file 41564_2021_980_MOESM2_ESM.pdf]

nature portfolio

Corresponding author(s): Nicholas Youngblut

Last updated by author(s): Aug 27, 2021

Reporting Summary

Nature Portfolio wishes to improve the reproducibility of the work that we publish. This form provides structure for consistency and transparency in reporting. For further information on Nature Portfolio policies, see our Editorial Policies and the Editorial Policy Checklist.

Statistics

For all statistical analyses, confirm that the following items are present in the figure legend, table legend, main text, or Methods section.

n/a	Confirmed	
☐	☒	The exact sample size (*n*) for each experimental group/condition, given as a discrete number and unit of measurement
☐	☒	A statement on whether measurements were taken from distinct samples or whether the same sample was measured repeatedly
☐	☒	The statistical test(s) used AND whether they are one- or two-sided<br>*Only common tests should be described solely by name; describe more complex techniques in the Methods section.*
☐	☒	A description of all covariates tested
☐	☒	A description of any assumptions or corrections, such as tests of normality and adjustment for multiple comparisons
☐	☒	A full description of the statistical parameters including central tendency (e.g. means) or other basic estimates (e.g. regression coefficient) AND variation (e.g. standard deviation) or associated estimates of uncertainty (e.g. confidence intervals)
☐	☒	For null hypothesis testing, the test statistic (e.g. *F*, *t*, *r*) with confidence intervals, effect sizes, degrees of freedom and *P* value noted<br>*Give P values as exact values whenever suitable.*
☒	☐	For Bayesian analysis, information on the choice of priors and Markov chain Monte Carlo settings
☒	☐	For hierarchical and complex designs, identification of the appropriate level for tests and full reporting of outcomes
☐	☒	Estimates of effect sizes (e.g. Cohen's *d*, Pearson's *r*), indicating how they were calculated

Our web collection on statistics for biologists contains articles on many of the points above.

Software and code

Policy information about availability of computer code

Data collection	All information on the data collection can be found in the manuscript (e.g., Methods and Supplementary Table 1) along with information at https://github.com/leylabmpi/16S-arc_vertebrate_paper. The following software was used: QIIME2 v2019.10, DADA2 v1.10.0, QIIME2 q2-feature-classifier v2019.10.0, iNEXT R package v2.0.19, zCompositions R package v1.3.3, MAFFT v7.310, metaSPAdes v3.12.0, minimap2 v2.20, Kraken2 v2.1.1, Bracken v2.6.2, Struo v0.1.7, Ecodist R package v2.0.5, Vegan R package v2.5-6 , phylosignal R package v1.3, PACo R package v0.4.2, APE R package v5.5, phytools R package v0.7-70, RRPP R package v0.6.2, Rphylopars R package v0.3.0, cooccur R package v1.3, igraph R package v.1.2.6, iTOL 6.3, tidygraph R package v1.2.0, ggraph R package v2.0.4, batchtools R package v0.9.13, clustermq v0.8.95.1, dplyr R package v1.0.1, tidyr R package v1.1.0, ggplot2 R package v3.3.2
Data analysis	All code required to reproduce the analysis can be found at https://github.com/leylabmpi/16S-arc_vertebrate_paper

For manuscripts utilizing custom algorithms or software that are central to the research but not yet described in published literature, software must be made available to editors and reviewers. We strongly encourage code deposition in a community repository (e.g. GitHub). See the Nature Portfolio guidelines for submitting code & software for further information.

Data

Policy information about availability of data

All manuscripts must include a data availability statement. This statement should provide the following information, where applicable:

- Accession codes, unique identifiers, or web links for publicly available datasets
- A description of any restrictions on data availability
- For clinical datasets or third party data, please ensure that the statement adheres to our policy

Genome Taxonomy Database (https://gtdb.ecogenomic.org/)

PRJEB40672
PRJEB38078
SILVA (https://www.arb-silva.de/)
BLAST nr (https://ftp.ncbi.nlm.nih.gov/blast/)
Struo GTDB-r95 (http://ftp.tue.mpg.de/ebio/projects/struo/GTDB_release95/)

Field-specific reporting

Please select the one below that is the best fit for your research. If you are not sure, read the appropriate sections before making your selection.

☐ Life sciences ☐ Behavioural & social sciences ☒ Ecological, evolutionary & environmental sciences

For a reference copy of the document with all sections, see nature.com/documents/nr-reporting-summary-flat.pdf

Field work, collection and transport

Field conditions	Samples were collected from many locations around the world at varying times of the year. See the supplemental metadata provided with the manuscript. Habitats include anthropogenic, cultivated, freshwater, grassland, saline water, terrestrial, and woodland. Ambient temperature and rainfall were not recorded.
Location	Sample=F14_Common_Bream,latitude=48.20833,longitude=16.373064,elevation=171m;Sample=F35_Red_Deer,latitude=48.20833,longitude=16.373064,elevation=171m;Sample=F36_Red_Deer,latitude=48.20833,longitude=16.373064,elevation=171m;Sample=F44_Fallow_Deer,latitude=48.20833,longitude=16.373064,elevation=171m;Sample=F45_Red_Deer,latitude=48.20833,longitude=16.373064,elevation=171m;Sample=F46_Red_Deer,latitude=48.20833,longitude=16.373064,elevation=171m;Sample=F47_Red_Deer,latitude=48.20833,longitude=16.373064,elevation=171m;Sample=F48_Red_Deer,latitude=48.20833,longitude=16.373064,elevation=171m;Sample=F53_Mouflon,latitude=48.20833,longitude=16.373064,elevation=171m;Sample=F66_Wild_Boar,latitude=48.20833,longitude=16.373064,elevation=171m;Sample=F68_Red_Deer,latitude=48.20833,longitude=16.373064,elevation=171m;Sample=F69_Red_Deer,latitude=48.20833,longitude=16.373064,elevation=171m;Sample=F70_Red_Deer,latitude=48.20833,longitude=16.373064,elevation=171m;Sample=F80_Red_Deer,latitude=48.20833,longitude=16.373064,elevation=171m;Sample=F90_Domestic_Dog,latitude=48.20833,longitude=16.373064,elevation=171m;Sample=X3_Alpine_Chamois,latitude=47.7779,longitude=13.23383,elevation=784m;Sample=X7_European_Otter,latitude=47.062,longitude=15.415,elevation=259m;Sample=X11_Onager,latitude=29.608056,longitude=52.524722,elevation=1544m;Sample=X13_Wolf,latitude=46.866667,longitude=14.116667,elevation=825m;Sample=X15_Tawny_Owl,latitude=48.20833,longitude=16.373064,elevation=171m;Sample=X16_Fat_Dormouse,latitude=48.20833,longitude=16.373064,elevation=171m;Sample=X22_European_Rabbit,latitude=48.20833,longitude=16.373064,elevation=171m;Sample=X23_Garden_Dormouse,latitude=48.20833,longitude=16.373064,elevation=171m;Sample=X31_Chicken,latitude=48.783333,longitude=15.066667,elevation=526m;Sample=X32_Greylag_Goose,latitude=48.783333,longitude=15.066667,elevation=526m;Sample=X33_Wild_Turkey,latitude=48.783333,longitude=15.066667,elevation=526m;Sample=X34_Mallard_Duck,latitude=48.783333,longitude=15.066667,elevation=526m;Sample=X40_Bactrian_Camel,latitude=43.707594,longitude=98.349609,elevation=1119m;Sample=X41_Pika,latitude=43.707594,longitude=98.349609,elevation=1119m;Sample=X42_Goitered_Gazelle,latitude=43.707594,longitude=98.349609,elevation=1119m;Sample=X43_European_Badger,latitude=48.20833,longitude=16.373064,elevation=171m;Sample=X60_European_Chub,latitude=48.2798373,longitude=15.411374,elevation=472m;Sample=F157a_European_Toad,latitude=48.333333,longitude=15.75,elevation=278m;Sample=X77_Onager,latitude=29.608056,longitude=52.524722,elevation=1544m;Sample=X85_Domestic_Dog,latitude=48.783333,longitude=15.066667,elevation=526m;Sample=X88_European_Rabbit,latitude=48.20833,longitude=16.373064,elevation=171m;Sample=X93_Red_Sheep,latitude=48.783333,longitude=15.066667,elevation=526m;Sample=X94_Mangalica,latitude=48.783333,longitude=15.066667,elevation=526m;Sample=X95_Meadow_Viper,latitude=47.5,longitude=19.05,elevation=104m;Sample=X101_Horse,latitude=48.783333,longitude=15.066667,elevation=526m;Sample=X102_Kulan,latitude=43.707594,longitude=98.349609,elevation=1119m;Sample=X109_Red_Fox,latitude=48.20833,longitude=16.373064,elevation=171m;Sample=X111_West_European_Hedgehog,latitude=47.5,longitude=19.05,elevation=104m;Sample=X116_Common_Kestrel,latitude=47.5,longitude=19.05,elevation=104m;Sample=X117_Long_eared_Owl,latitude=47.5,longitude=19.05,elevation=104m;Sample=X119_Ural_Owl,latitude=48.004722,longitude=15.167778,elevation=339m;Sample=X121_Raccoon_Dog,latitude=48.333333,longitude=15.75,elevation=278m;Sample=X122_Wild_Boar,latitude=47.84637,longitude=16.52796,elevation=168m;Sample=X123_Alpine_Chamois,latitude=47.04266,longitude=12.89106,elevation=1745m;Sample=X125_Common_Carp,latitude=48.7564,longitude=16.501147,elevation=200m;Sample=X127_Alpine_Marmot,latitude=47.0267064,longitude=12.7899939,elevation=1683m;Sample=X128_Alpine_Marmot,latitude=47.0267064,longitude=12.7899939,elevation=1683m;Sample=X129_Alpine_Ibex,latitude=47.066667,longitude=12.783333,elevation=2278m;Sample=X130_Alpine_Ibex,latitude=47.0855556,longitude=12.723333,elevation=2356m;Sample=X131_Rock_Ptarmigan,latitude=47.05,longitude=13.39999,elevation=2220m;Sample=X133_Domestic_Goat,latitude=47.5,longitude=16.416667,elevation=335m;Sample=X134_Domestic_Goat,latitude=47.5,longitude=16.416667,elevation=335m;Sample=X135_Horse,latitude=47.5,longitude=16.416667,elevation=335m;Sample=X157_Grey_Heron,latitude=48.333333,longitude=15.75,elevation=278m;Sample=X158_White_tailed_Eagle,latitude=48.33,longitude=16.868056,elevation=146m;Sample=X137_Gaur,latitude=11.423333,longitude=107.428611,elevation=120m;Sample=X138_Gaur,latitude=11.423333,longitude=107.428611,elevation=120m;Sample=X139_Sambar,latitude=11.423333,longitude=107.428611,elevation=120m;Sample=X140_Sambar,latitude=11.423333,longitude=107.428611,elevation=120m;Sample=X164_Greylag_Goose,latitude=54.516667,longitude=8.636944,elevation=minus2m;Sample=X170_Tawny_Owl,latitude=48.20833,longitude=16.373064,elevation=171m;Sample=X172_European_Greenfinch,latitude=47.5,longitude=19.05,elevation=104m;Sample=X174_Great_Tit,latitude=47.5,longitude=19.05,elevation=104m;Sample=X153_Bank_Vole,latitude=48.20833,longitude=16.373064,elevation=171m;Sample=X146_Cattle,latitude=50.59549,longitude=12.6386475,elevation=468m;Sample=X194_Agile_Frog,latitude=48.20833,longitude=16.373064,elevation=171m;Sample=X154_Tree_Shrew,latitude=48.20833,longitude=16.373064,elevation=171m;Sample=X141_Red_cheeked_Gibbon,latitude=11.423333,longitude=107.428611,elevation=120m;Sample=X179_Roach,latitude=48.236304,longitude=15.333137,elevation=207m;Sample=X195_Domestic_Cat,latitude=48.20833,longitude=16.373064,elevation=171m;Sample=X203_Red_Deer,latitude=47.0177778,longitude=12.780833,elevation=1868m;Sample=X206_Red_Sheep,latitude=47.726944,longitude=16.081667,elevation=367m;Sample=X212_Goose,latitude=47.5,longitude=16.416667,elevation=335m;Sample=X213_Goose,latitude=47.5,longitude=16.416667,elevation=335m;Sample=X214_Goose,latitude=47.5,longitude=16.416667,elevation=335m;Sample=X215_Goose,latitude=47.5,longitude=16.416667,elevation=335m;Sample=X218_Goose,latitude=47.5,longitude=16.416667,elevation=335m;Sample=X219_Goose,latitude=47.5,longitude=16.416667,elevation=335m;Sample=X247_Common_Hamster,latitude=48.410833,longitude=15.610278,elevation=195m;Sample=X252_Eurasian_Lynx,latitude=48.226667,longitude=14.179444,elevation=293m;Sample=X320_Red_Deer,latitude=48.20833,longitude=16.373064,elevation=171m;Sample=X233_Aesculapian_Snake,latitude=48.20833,longitude=16.373064,elevation=171m;Sample=X236_Pygmy_Slow_Loris,latitude=20.316667,longitude=105.608333,elevation=306m;Sample=X237_Red_shanked_Douc_Langur,latitude=20.316667,longitude=105.608333,elevation=306m;Sample=X238_Hanuman_Langur,latitude=20.316667,longitude=105.608333,elevation=306m;Sample=X240_Southern_White_cheeked_Gibbon,latitude=20.316667,longitude=105.608333,elevation=306m;Sample=X241_Italian_wall_lizard,latitude=45.25,longitude=15.466667,elevation=241m;Sample=X242_Dalmatian_Tortoise,latitude=45.25,longitude=15.466667,elevation=241m;Sample=X230_Carrion_Crow,latitude=48.333333,longitude=15.75,elevation=278m;Sample=X234_Beech_Marten,latitude=48.20833,longitude=16.373064,elevation=171m;Sample=X259_Mute_Swan,latitude=47.5,longitude=19.05,elevation=104m;Sample=X260_Blackbird,latitude=47.5,longitude=19.05,elevation=104m;Sample=X265_Western_Marsh_Harrier,latitude=48.333333,longitude=15.75,elevation=278m;Sample=X266_Western_Marsh_Harrier,latitude=48.333333,longitude=15.75,elevation=278m;Sample=X268_Rook,latitude=47.793889,longitude=16.495833,elevation=168m;Sample=X270_Common_Pheasant,latitude=48.477146,longitude=16.2079188,elevation=224m;Sample=X271_Common_Pheasant,latitude=48.477146,longitude=16.2079188,elevation=224m;Sample=X279_Western_Grey_Kangaroo,latitude=-35.833333,longitude=137.25,elevation=157m;Sample=X280_Western_Grey_Kangaroo,latitude=-35.833333,longitude=137.25,elevation=157m;Sample=X281_Tammar_Wallaby,latitude=-35.833333,longitude=137.25,elevation=157m;Sample=X283_Koala,latitude=-34.95,longitude=138.6833

Ecological, evolutionary & environmental sciences study design

All studies must disclose on these points even when the disclosure is negative.

Study description	We studied the gut microbiome of wild and captive animals. The major treatments were animal diet and evolutionary history. Other covariates included captivity status (wild vs captive) and sample type (gut contents or feces). Treatment factors and covariates include: host evolutionary history, host diet, host habitats, geographic distance between sampling locations, sample type (feces versus gut contents, and host captive/wild status. The dataset is hierarchically structured via host and microbial taxonomy and evolutionary history. The total number of experimental units (GI samples) is 311, but only 185 in the 16S rRNA sequence dataset. The replicates per treatment, when considering host species as treatments, varied from 1 to 12.
Research sample	The feces (or gut contents) from an individual animal was considered a single sample. We only used one sample per animal species for each hypothesis test, due to a lack of data on intra-species host evolutionary history. The gender ratio of individuals in which sex was known (n = 79) was 41:38 (m:f). The age range for the individual in which age is know is 2-10 years.
Sampling strategy	Samples were collected by wildlife biologists. No sample size calculation was performed. Sample sizes were chosen based on sample availability. The sample sizes were sufficient to decouple the association of host evolutionary history and diet with archaeal diversity, which is the major goal of this work.
Data collection	Sampling metadata was collected by sampling teams at the respective locations and reported by email to the study team who aggregated metadata in spreadsheets, adding appropriate additional data from literature sources and databases (diet data, host taxonomy data, etc.). Sample data was collected by the following persons: Mario Baldi, School of Veterinary Medicine, Universidad Nacional de Costa Rica; Wolfgang Vogl and Frank Radon, Konrad Lorenz Institute of Ethology and Biological Station Illmitz; Endre Sós and Viktor Molnár, Budapest Zoo; Ulrike Streicher, Conservation and Wildlife Management Consultant, Vietnam; Katharina Mahr, Konrad Lorenz Institute of Ethology, University of Veterinary Medicine Vienna and Flinders University Adelaide, South Australia; Peggy Rismiller, Pelican Lagoon Research Centre, Australia; Rob Deaville, Institute of Zoology, Zoological Society of London; Alex Lécu, Muséum National d'Histoire Naturelle and Paris Zoo; Danny Govender and Emily Lane, South African National Parks, Sanparks; Fritz Reimoser, Research Institute of Wildlife Ecology, University of Veterinary Medicine Vienna; Anna Kübber-Heiss and Team, Pathology, Research Institute of Wildlife Ecology, University of Veterinary Medicine Vienna; Nikolaus Eisank, Nationalpark Hohe Tauern, Kärnten; Attila Hettyey and Yoshan Moodley, Konrad Lorenz Institute of Ethology, University of Veterinary Medicine Vienna; Mansour El-Matbouli and Oskar Schachner, Clinical Unit of Fish Medicine, University of Veterinary Medicine; Barbara Richter, Institute of Pathology and Forensic Veterinary Medicine, University of Veterinary Medicine Vienna; Hanna Vielgrader and Zoovet Team, Schönbrunn Zoo; Reinhard Pichler, Herberstein Zoo. Freek Venter, South African National Parks and the National Zoological Gardens of South Africa. DNA concentrations were measured using a PicoGreen reagent (Thermo-Fisher, Vienna, Austria) using a Anthos Zenyth fluorescence plate reader (Biochrom, Cambridge UK).
Timing and spatial scale	Sampling was conducted from February 2009 and March 2014. Samples originated predominantly from Central Europe (Austria and neighboring countries). However, in order to cover as much vertebrate diversity as possible, many samples were also taken from other countries around the world (see the metadata provided with the manuscript). The frequency and periodicity of sampling was based on sample manpower availability.
Data exclusions	A small subset of samples were excluded from all analyses of the 16S rRNA sequence data due to not enough sampling depth of the microbiome. These exclusion criteria were not pre-established, but such exclusions are standard for microbiome data.
Reproducibility	We assessed each question with multiple analyses and compared our results to previous studies in order to assess reproducibility. Experimental replication was not performed.
Randomization	We utilize a sensitivity method of randomly subsampling one individual sample per species for each hypothesis test, repeating this procedure a total of 100 times, and using the 95% quartile of significance values for each individual subsample to assess overall significance. This allowed us to assess how sensitive our analysis was to intra-species heterogeneity, which would be missed if we had simply used one randomly subsample sample per animal species.
Blinding	No blinding was used, given that this study is not set up like a clinical trial; we are not testing control versus treatment as done with a drug trial, or similar.

Did the study involve field work? ☒ Yes ☐ No

33,elevation=388m;Sample=X285_Short_beaked_Echidna,latitude=-35.833333,longitude=137.25,elevation=157m;Sample=X286_Short_beaked_Echidna,latitude=-35.833333,longitude=137.25,elevation=157m;Sample=X287_Common_Brushtail,latitude=-34.929,longitude=138.601,elevation=45m;Sample=X288_Common_Brushtail,latitude=-34.929,longitude=138.601,elevation=45m;Sample=X289_Eastern_Grey_Kangaroo,latitude=-30.000232,longitude=136.209155,elevation=132m;Sample=X290_Eastern_Grey_Kangaroo,latitude=-30.000232,longitude=136.209155,elevation=132m;Sample=X292_Central_Bearded_Dragon,latitude=-35.63,longitude=138.503611,elevation=69m;Sample=X305_European_Hare,latitude=48.7564,longitude=16.501147,elevation=200m;Sample=X326_Silver_Bream,latitude=48.721773,longitude=16.193848,elevation=190m;Sample=X327_Silver_Bream,latitude=48.721773,longitude=16.193848,elevation=190m;Sample=X328_Prussian_Carp,latitude=48.721773,longitude=16.193848,elevation=190m;Sample=X330_Ide,latitude=48.721773,longitude=16.193848,elevation=190m;Sample=X333_Gray_Seal,latitude=54.958004,longitude=-1.351318,elevation=0m;Sample=X336_Sei_Whale,latitude=55.289869,longitude=-1.564178,elevation=0m;Sample=X338_White_beaked_Dolphin,latitude=51.395136,longitude=1.380501,elevation=0m;Sample=X339_White_beaked_Dolphin,latitude=52.375861,longitude=1.712236,elevation=0m;Sample=X340_Western_Lowland_Gorilla,latitude=0.219726,longitude=14.853516,elevation=414m;Sample=X341_Western_Lowland_Gorilla,latitude=0.219726,longitude=14.853516,elevation=414m;Sample=X344_Indian_Rhinoceros,latitude=27.529131,longitude=84.354205,elevation=197m;Sample=X349_Smooth_Newt,latitude=48.333333,longitude=15.75,elevation=278m;Sample=X350_Smooth_Newt,latitude=48.333333,longitude=15.75,elevation=278m;Sample=X351_Grass_Snake,latitude=48.20833,longitude=16.373064,elevation=171m;Sample=X352_Kinkajou,latitude=12.822514,longitude=-84.100342,elevation=45m;Sample=X353_Raccoon,latitude

=9.96304,longitude=-84.04823,elevation=1237m;Sample=X356_Northern_Tamandua,latitude=6.489983,longitude=-75.19043,elevation=1980m;Sample=X357_Northern_Tamandua,latitude=6.489983,longitude=-75.19043,elevation=1980m;Sample=X359_Brown_throated_Sloth,latitude=12.822514,longitude=-84.100342,elevation=45m;Sample=X360_White_tailed_Deer,latitude=9.748917,longitude=-83.753428,elevation=1403m;Sample=X363_Desmarests_Spiny_Pocket_Mouse,latitude=9.748917,longitude=-83.753428,elevation=1403m;Sample=X368_European_Ground_Squirrel,latitude=48.20833,longitude=16.373064,elevation=171m;Sample=X369_Koala,latitude=48.20833,longitude=16.373064,elevation=171m;Sample=X370_African_Bush_Elephant,latitude=48.20833,longitude=16.373064,elevation=171m;Sample=X371_Linnaeus_Two_toed_Sloth,latitude=48.20833,longitude=16.373064,elevation=171m;Sample=X372_Emu,latitude=48.20833,longitude=16.373064,elevation=171m;Sample=X376_Kulan,latitude=45.4,longitude=92.9,elevation=1345m;Sample=X378_Fat_Sand_Rat,latitude=48.20833,longitude=16.373064,elevation=171m;Sample=X380_Wild_Boar,latitude=47.5,longitude=16.416667,elevation=335m;Sample=X381_Wild_Boar,latitude=47.5,longitude=16.416667,elevation=335m;Sample=X382_Arctic_Wolf,latitude=47.224,longitude=15.812,elevation=423m;Sample=X384_Eurasian_Lynx,latitude=47.224,longitude=15.812,elevation=423m;Sample=X389_Indian_Rhinoceros,latitude=48.20833,longitude=16.373064,elevation=171m;Sample=X390_European_Ground_Squirrel,latitude=48.20833,longitude=16.373064,elevation=171m;Sample=X391_European_Ground_Squirrel,latitude=48.2276,longitude=14.024048,elevation=354m;Sample=X394_Przewalski_horse,latitude=45.4,longitude=92.9,elevation=1345m;Sample=X395_Przewalski_horse,latitude=47.224,longitude=15.812,elevation=423m;Sample=X396_Morelets_crocodile,latitude=48.20833,longitude=16.373064,elevation=171m;Sample=X403_Chacma_Baboon,latitude=-25.119855,longitude=31.91541,elevation=176m;Sample=X404_Brown_Greater_Galago,latitude=-24.995833,longitude=31.591944,elevation=288m;Sample=X405_Nile_Crocodile,latitude=-23.231251,longitude=30.492554,elevation=510m;Sample=X407_African_Bush_Elephant,latitude=-24.995833,longitude=31.591944,elevation=288m;Sample=X408_African_Bush_Elephant,latitude=-24.995833,longitude=31.591944,elevation=288m;Sample=X409_Impala,latitude=-24.995833,longitude=31.591944,elevation=288m;Sample=X410_Impala,latitude=-24.995833,longitude=31.591944,elevation=288m;Sample=X411_Leopard,latitude=-24.439148,longitude=31.438065,elevation=418m;Sample=X414_Vervet_Monkey,latitude=-24.995833,longitude=31.591944,elevation=288m;Sample=X415_White_Rhinoceros,latitude=-23.231251,longitude=30.492554,elevation=510m;Sample=X416_White_Rhinoceros,latitude=-23.231251,longitude=30.492554,elevation=510m;Sample=X417_Giraffe,latitude=1.230374,longitude=38.583984,elevation=345m;Sample=X418_Giraffe,latitude=1.230374,longitude=38.583984,elevation=345m;Sample=X423_Quagga,latitude=-2.416276,longitude=34.650879,elevation=1527m;Sample=X424_Quagga,latitude=-2.416276,longitude=34.650879,elevation=1527m;Sample=X421_Common_Hippopotamus,latitude=-2.416276,longitude=34.650879,elevation=1527m;Sample=X422_Common_Hippopotamus,latitude=-2.416276,longitude=34.650879,elevation=1527m;Sample=X419_African_Buffalo,latitude=-2.416276,longitude=34.650879,elevation=1527m;Sample=X420_Cattle,latitude=-2.416276,longitude=34.650879,elevation=1527m;Sample=X426_Ural_Owl,latitude=48.20833,longitude=16.373064,elevation=171m;Sample=X427_Great_Spotted_Woodpecker,latitude=48.20833,longitude=16.373064,elevation=171m;Sample=X428_African_Bush_Elephant,latitude=48.20833,longitude=16.373064,elevation=171m;Sample=X429_Giraffe,latitude=48.20833,longitude=16.373064,elevation=171m;Sample=X430_Giraffe,latitude=48.20833,longitude=16.373064,elevation=171m;Sample=X431_Quagga,latitude=47.224,longitude=15.812,elevation=423m;Sample=X432_Vervet_Monkey,latitude=47.224,longitude=15.812,elevation=423m;Sample=X434_Blue_Wildebeest,latitude=47.224,longitude=15.812,elevation=423m;Sample=X435_Bactrian_Camel,latitude=47.224,longitude=15.812,elevation=423m;Sample=X66_Mouflon,latitude=48.20833,longitude=16.373064,elevation=171m;Sample=X221_Alpine_Ibex,latitude=47.7594088,longitude=13.060725,elevation=436m;Sample=X73_One_humped_Camel,latitude=48.214922,longitude=16.936046,elevation=158m;Sample=X67_European_Roe,latitude=48.20833,longitude=16.373064,elevation=171m;Sample=X96_European_Hare,latitude=47.897778,longitude=16.908889,elevation=126m

Access & import/export

Wildlife biologists, who were conducting long-term research on the respective species in its habitat, ensured that sampling guidelines and restrictions were adhered to, where these were applicable. Samples were collected by the following persons:
Mario Baldi, School of Veterinary Medicine, Universidad Nacional de Costa Rica; Wolfgang Vogl and Frank Radon, Konrad Lorenz Institute of Ethology and Biological Station Illmitz; Endre Sós and Viktor Molnár, Budapest Zoo; Ulrike Streicher, Conservation and Wildlife Management Consultant, Vietnam; Katharina Mahr, Konrad Lorenz Institute of Ethology, University of Veterinary Medicine Vienna and Flinders University Adelaide, South Australia; Peggy Rismiller, Pelican Lagoon Research Centre, Australia; Rob Deaville, Institute of Zoology, Zoological Society of London; Alex Lécu, Muséum National d'Histoire Naturelle and Paris Zoo; Danny Govender and Emily Lane, South African National Parks, Sanparks; Fritz Reimoser, Research Institute of Wildlife Ecology, University of Veterinary Medicine Vienna; Anna Kübber-Heiss and Team, Pathology, Research Institute of Wildlife Ecology, University of Veterinary Medicine Vienna; Nikolaus Eisank, Nationalpark Hohe Tauern, Kärnten; Attila Hettyey and Yoshan Moodley, Konrad Lorenz Institute of Ethology, University of Veterinary Medicine Vienna; Mansour El-Matbouli and Oskar Schachner, Clinical Unit of Fish Medicine, University of Veterinary Medicine; Barbara Richter, Institute of Pathology and Forensic Veterinary Medicine, University of Veterinary Medicine Vienna; Hanna Vielgrader and Zoovet Team, Schönbrunn Zoo; Reinhard Pichler, Herberstein Zoo. Freek Venter, South African National Parks and the National Zoological Gardens of South Africa. Most of the sampling did not require permits, since only non-invasive, fecal samples were collected. The City of Vienna issued a permit for the capture and sampling wild mice (issuing authority: Municipal department 22 of the City of Vienna, date of issue April 6 2011, permit number MA 22 - 229/2011). The South African National Parks organization issued a permit for collecting and exporting fecal material from the Park grounds (issuing authority: South African National Parks, date of issue November 18th 6 2013, reference number REISHG 1158)

Disturbance

No disturbance was caused.

Reporting for specific materials, systems and methods

We require information from authors about some types of materials, experimental systems and methods used in many studies. Here, indicate whether each material, system or method listed is relevant to your study. If you are not sure if a list item applies to your research, read the appropriate section before selecting a response.

Materials & experimental systems

n/a	Involved in the study
☒	☐ Antibodies
☒	☐ Eukaryotic cell lines
☒	☐ Palaeontology and archaeology
☒	☐ Animals and other organisms
☒	☐ Human research participants
☒	☐ Clinical data
☒	☐ Dual use research of concern

Methods

n/a	Involved in the study
☒	☐ ChIP-seq
☒	☐ Flow cytometry
☒	☐ MRI-based neuroimaging
